# Effect of counter-ion on packing and crystal density of 5,5′-(3,3′-bi[1,2,4-oxa­diazole]-5,5′-di­yl)bis­(1*H*-tetra­zol-1-olate) with five different cations

**DOI:** 10.1107/S205698901800364X

**Published:** 2018-03-09

**Authors:** Ian D. Giles, Alan J. DeHope, Nathaniel B. Zuckerman, Damon A. Parrish, Philip F. Pagoria

**Affiliations:** a CBMSE, Code 6910, Naval Research Laboratory, Washington, DC 20375, USA; b Lawrence Livermore National Laboratory, 7000 East Ave, Mail Stop L-282, Livermore, CA 94550, USA

**Keywords:** crystal structure, tetra­zole, oxa­diazole, tri­amino­guandidinium, hydrazinium, hydroxyl­ammonium, amino­guanidinium, 5-amino-1*H*-tetra­zol-4-ium, energetic materials

## Abstract

In energetic materials, the crystal density is an important parameter that affects the performance of the material. When making ionic energetic materials, the choice of counter-ion can have detrimental or beneficial effects on the packing, and therefore the density, of the resulting energetic crystal. Presented herein are a series of five ionic energetic crystals, all containing the dianion 5,5′-(3,3′-bi[1,2,4-oxa­diazole]-5,5′-di­yl)bis­(1*H*-tetra­zol-1-olate), with the following cations: hydrazinium (**1**) (2N_2_H_5_^+^·C_6_N_12_O_4_^2−^), hydroxyl­ammonium (**2**) 2NH_4_O^+^·C_6_N_12_O_4_^2−^ [Pagoria *et al..* (2017). *Chem. Heterocycl. Compd*, **53**, 760–778; included for comparison], di­methyl­ammonium (**3**) (2C_2_H_8_N^+^·C_6_N_12_O_4_^2−^), 5-amino-1*H*-tetra­zol-4-ium (**4**) (2CH_4_N_5_^+^·C_6_N_12_O_4_^2−^·4H_2_O), and amino­guanidinium (**5**) (2CH_7_N_4_^+^·C_6_N_12_O_4_^2−^). Both the supra­molecular inter­actions and the sterics of the cation play a role in the density of the resulting crystals, which range from 1.544 to 1.873 Mg m^−1^. In **5**, the tetra­zolate ring is disordered over two positions [occupancy ratio 0.907 (5):0.093 (5)] due to a 180° rotation in the terminal tetra­zole rings.

## Abstract 2

In energetic materials, the crystal density is an important parameter that affects the performance of the material. When making ionic energetic materials, the choice of counter-ion can have detrimental or beneficial effects on the packing, and therefore the density, of the resulting energetic crystal. Presented herein are a series of five ionic energetic crystals, all containing the 5,5′-(3,3′-bi[1,2,4-oxa­diazole]-5,5′-di­yl)bis­(1*H*-tetra­zol-1-olate) dianion.

## Chemical context   

One of the critical parameters directly related to the performance of an energetic material, specifically its detonation velocity, is the density of the material (Ma *et al.*, 2014 ▸; Akhavan, 2011 ▸). This is an important consideration when designing energetic materials that incorporate counter-ions into their structures, since these counter-ions can, through supra­molecular inter­actions, aid or disrupt effective packing of the mol­ecule in question. Presented herein are the structures of a single energetic mol­ecule, 5,5′-(3,3′-bi[1,2,4-oxa­diazole]-5,5′-di­yl)bis­(1*H*-tetra­zol-1-olate), as salts of five different cations: hydrazinium (**1**), hydroxyl­ammonium (**2**) (Pagoria *et al.*, 2017 ▸, included for comparison), di­methyl­ammonium (**3**), 5-amino-1*H*-tetra­zol-4-ium (**4**), and amino­guanidinium (**5**). As a result of the variety of cation structures and inter­molecular inter­actions, each exhibits subtly different crystal packing, which affects the resulting density. The mol­ecule of inter­est, however, only exhibits minor changes in bond distances depending on the cation.

## Structural commentary   

The primary mol­ecule, 5,5′-(3,3′-bi[1,2,4-oxa­diazole]-5,5′-di­yl)bis­(1*H*-tetra­zol-1-olate), is comprised of four penta­nuclear rings, with two 1,2,4-oxa­diazole rings linked together through the 5-position carbon atom, and the tetra­zol-1-olate rings linked at the 5-position carbon atom to each 1,2,4-oxa­diazole ring at the 3-position carbon.
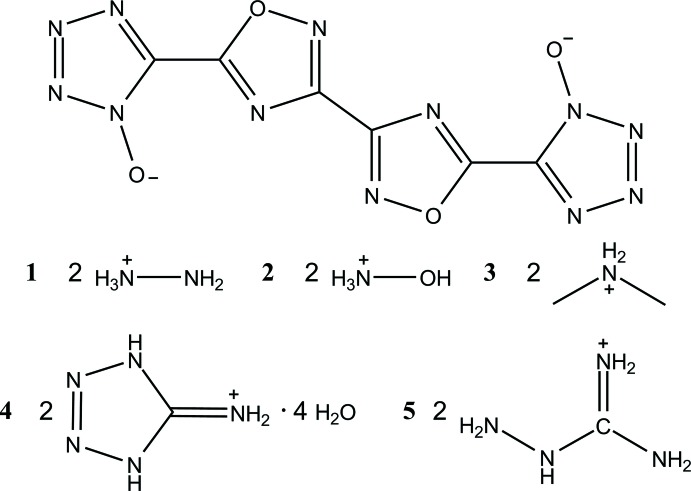


**Figure 1:**
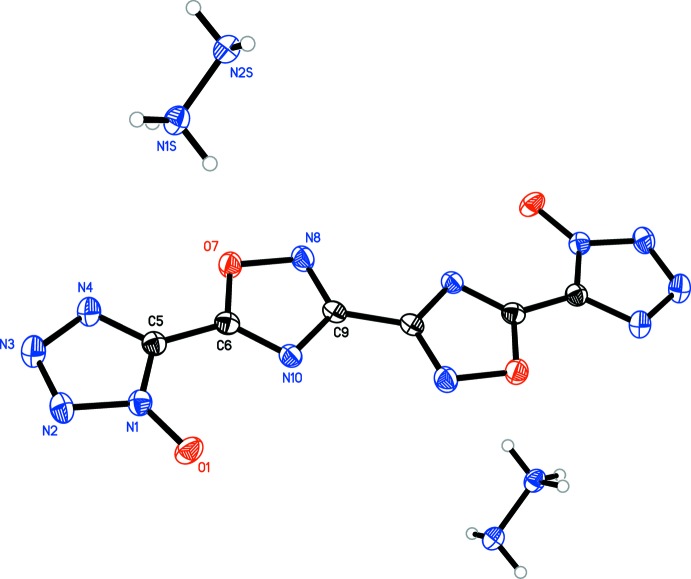
Mol­ecular structure of **1**, showing the atom-labeling scheme. Displacement ellipsoids are drawn at the 50% probability level.

**Figure 2:**
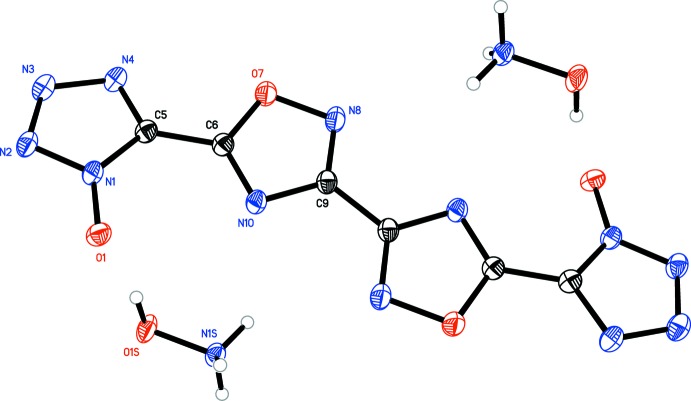
Mol­ecular structure of **2**, showing the atom-labeling scheme. Displacement ellipsoids are drawn at the 50% probability level.

**Figure 3:**
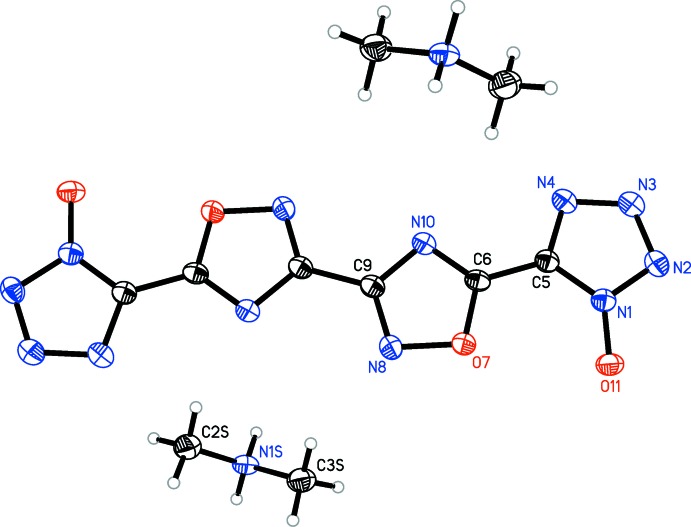
Mol­ecular structure of **3**, showing the atom-labeling scheme. Displacement ellipsoids are drawn at the 50% probability level.

**Figure 4:**
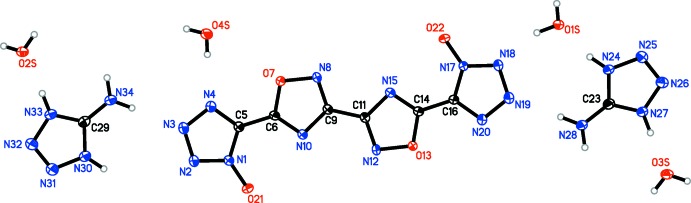
Mol­ecular structure of **4**, showing the atom-labeling scheme. Displacement ellipsoids are drawn at the 50% probability level.

In each structure, the oxa­diazole oxygen atoms are on opposite sides. For **1**, **2**, **3**, and **5** (Figs. **1**–**3** ▸ ▸ ▸, **5** ▸), the oxa­diazole rings are coplanar with one another, with the N8—C9—C9′—N8′ torsion angles constrained to 180°. Only slight deviation from coplanarity is seen in **4** (Fig. 4 ▸), with the N8—C9—C11—N12 torsion angles measuring 179.34 (16)°. Coincidently, **4** is the only structure in which the primary mol­ecule does not reside on an inversion center. For all structures, except **3**, the tetra­zolate ring is oriented such that the oxygen atoms of the oxa­diazole and tetra­zolate are on opposite sides, although **4** has a minor component of disorder [9.3 (4)%] in which one tetra­zolate is flipped by 180°. The N4—C5—C6—N10 torsion angles for **1** [174.25 (13)°], **4** [179.82 (16)°, N20—C16—C14—N15 angle is 176.68 (16)°], and **5** [N4*A*—C5*A*—C6—N10, 174.8 (5)°] show only slight deflections from coplanar, while in **2** [168.63 (15)°], the deflection is more pronounced. In structure **3**, the N4—C5—C6—N10 dihedral angle is 2.38 (19)°, showing only a slight deviation from coplanarity, despite the proximity of the two electronegative oxygen atoms.

In all five structures, the tetra­zolate C—N and N—N bond distances [ranging from 1.328 (5) to 1.351 (2) Å and 1.3170 (17) to 1.3455 (16) Å, respectively] suggest a delocalized aromatic system rather than discrete single and double bonds (Allen *et al.*, 1987 ▸). The oxa­diazole N—O, C—O, and C—N bond distances, however, suggest discrete single and double bonds. The N—O and C—O bonds range from 1.4033 (16) to 1.4115 (14) Å and 1.3391 (18) to 1.3468 (18) Å, respectively, suggesting single bonds between these atoms. The C—N bond opposite the oxygen atom ranges from 1.3671 (16) to 1.3755 (19) Å, also indicative of a single bond. The remaining C—N bonds range from 1.294 (2) to 1.309 (2) Å, typical for double bonds between these atoms. The central oxa­diazole–oxa­diazole C—C bond [ranging from 1.459 (3) to 1.465 (4) Å] and the C—C bonds linking the oxa­diazole rings to the tetra­zolate rings [ranging from 1.432 (2) to 1.447 (2) Å] are typical for C—C single bonds between non-fused heterocycles (Allen *et al.*, 1987 ▸).

Bond distances in the complex cations are typical for each. In **1**, the hydrazinium N—N bond distance of 1.4476 (16) Å matches the distance of 1.45 Å seen in hydrazinium chloride (Sakurai & Tomiie, 1952 ▸). In **2**, the hydroxyl­ammonium N—O bond distance of 1.4087 (16) Å matches the distance of 1.41 Å seen for hydroxyl­ammonium perchlorate (Dickens, 1969 ▸). In **3**, the di­methyl­ammonium C—N distances of 1.4767 (18) and 1.4780 (17) Å are consistent, albeit on the low side, with those reported for di­alkyl­ammonium ions, on average 1.494 (16) Å (Allen *et al.*, 1987 ▸). In **4**, the bond distances of 5-amino-1*H*-tetra­zol-4-ium are consistent with those seen in 5-amino-1*H*-tetra­zol-4-ium nitrate [bond type, distances (reference distances)]: C—N_amino_, 1.320 (2) and 1.314 (2) Å (1.308 Å); C—N_ring_, 1.334 (2) to 1.338 (2) Å (1.334 to 1.342 Å); C—N(H)—N=N, 1.357 (2) to 1.366 (2) Å (1.363 to 1.366 Å); N(H)—N=N—N(H), 1.272 (2) and 1.269 (2) Å (1.268 Å; von Denffer *et al.*, 2005 ▸). In **5**, the bond distances seen for the amino­guanidinium cation are consistent with those seen in amino­guandinium nitrate and are as follows [bond type, distances (reference distances)]: C—NH_2_, 1.309 (3) and 1.320 (3) Å (1.312 and 1.320 Å); C—N(H)(NH_2_), 1.337 (3) Å (1.328 Å); and N(H)—NH_2_, 1.420 (3) Å (1.399 Å; Akella & Keszler, 1994 ▸).

## Supra­molecular features   

Packing of the energetic mol­ecules will be described in four terms, following the example in Ma *et al.* (2014 ▸): sheet-like (with all mol­ecules parallel to one another), wavelike (with two mol­ecular planes that are not parallel to one another, but without inter­molecular crossing), crossing (same as wavelike but with inter­molecular crossing), and mixing (with mol­ecular planes that do not fit in the prior three categories).

**Figure 6:**
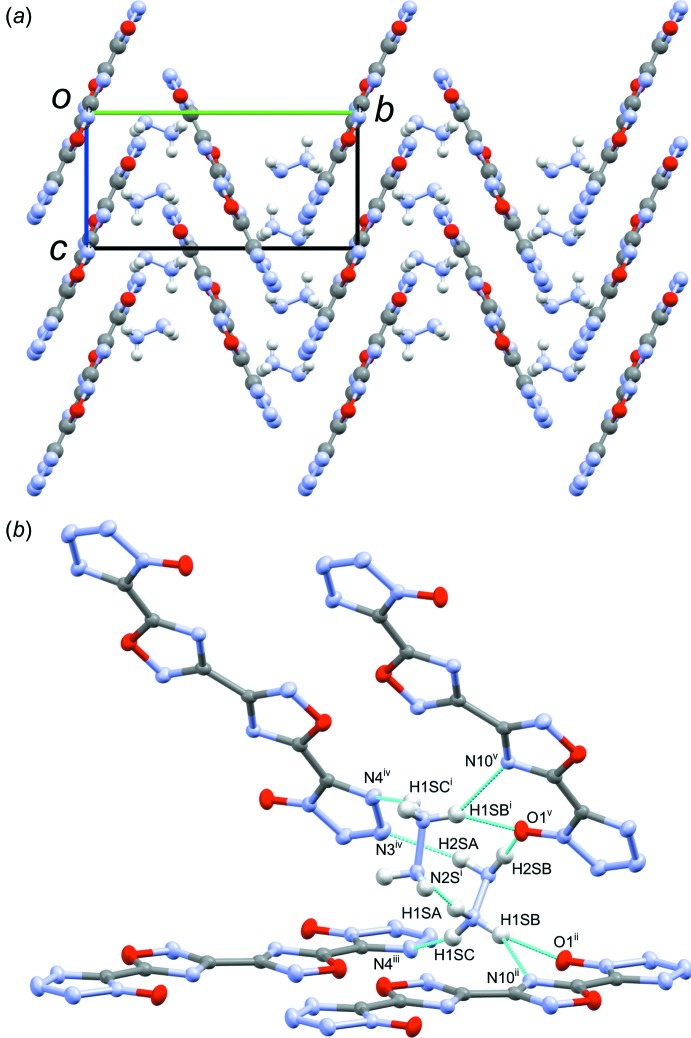
(*a*) Wavelike packing of **1** as seen down the *a*-axis, showing the opposing columns of the dianion with hydrazinium occupying gaps between the columns, and (*b*) view highlighting the hydrogen-bonding network (inter­molecular contacts) between the dianions and hydrazinium cations, and between the two hydrazinium cations. [Symmetry codes: (i) *x*, −*y* + 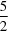, *z* + 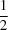; (ii) −*x* + 2, −*y* + 2, −*z* + 1; (iii) −*x* + 1, −*y* + 2, −*z* + 1; (iv) −*x* + 1, *y* + 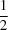, −*z* + 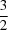; (v) −*x* + 2, *y* + 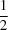, −*z* + 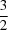.]

Structure **1**, space group *P*2_1_/*c*, packs in a wavelike pattern consisting of alternating columns of 5,5′-(3,3′-bi[1,2,4-oxa­diazole]-5,5′-di­yl)bis­(1*H*-tetra­zol-1-olate) (dianion) with the N2—N3 bond of one dianion over the tetra­zolate ring of the dianion in the neighboring column (Fig. 6 ▸ *a*). Hydrazinium ions occupy the gaps between neighboring coplanar dianions along the *b*-axis, above the plane of the mol­ecules. One hydrazinium forms a hydrogen-bonded network linking the neighboring intra­sheet dianions through the tetra­zolate oxygen, tetra­zolate N4 atom, and the NH_3_ portion of hydrazinium. Additionally, hydrogen bonds form between the NH_2_ portion of hydrazin­ium, the tetra­zolate oxygen atom, and the tetra­zolate N3 atom of neighboring dianions. An additional hydrogen bond connects the NH_3_ of one hydrazinium with the NH_2_ portion of the symmetry-related hydrazinium ion (Fig. 6 ▸ *b*, Table 1 ▸). Inter­molecular π–π stacking is limited in this structure, with tetra­zolate–oxa­diazole centroid_N1–N4/C5_–centroid_C6/O7/N8/C9/N10_ distances of 4.06 (2) and 4.01 (2) Å. The tetra­zolate oxygen atom forms an anion–π inter­action with the oxa­diazole ring of a neighboring dianion, with an O1-to-centroid_C6/O7/N8/C9/N10_ close contact of 2.98 (2) Å at an O1–centroid_C6/O7/N8/C9/N10_–O7 angle of 92.3 (2)° (Schottel, *et al.*, 2008 ▸).

**Figure 7:**
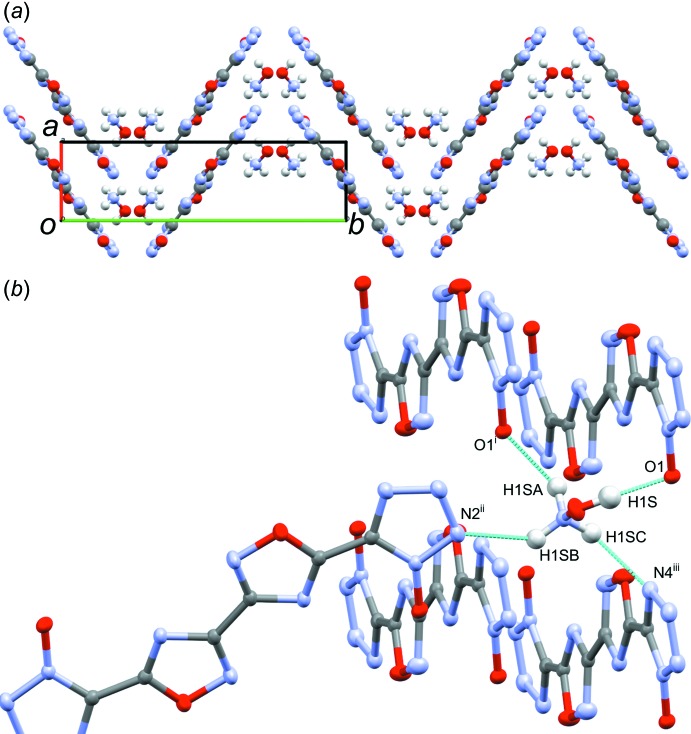
(*a*) Wavelike packing of **2** as seen down the *c*-axis, showing the opposing columns of dianion with hydroxyl­ammonium occupying the space between the columns, and (*b*) view highlighting the hydrogen-bonding network (inter­molecular contacts) between hydroxyl­ammonium cation and the dianions. [Symmetry codes: (i) *x* − 1, *y*, *z*; (ii) *x* − 1, −*y* + 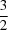, *z* + 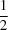; (iii) *x*, *y*, *z* + 1.]

Structure **2**, space group *P*2_1_/*c*, packs in a similar wavelike pattern as **1**; however, the N2—N3 bond of one dianion does not inter­act with the ring of neighboring dianions (Fig. 7 ▸ *a*). Additionally, the opposing columns are staggered with respect to one another. The hydroxyl­ammonium cations occupy the space formed where three dianion columns meet, above the dianion planes. The arrangement of the dianions in the peaks and troughs of the packing is dictated by the hydrogen bonds between the hydroxyl­ammonium hydroxyl group and the tetra­zolate oxygen atom, and those between the hydroxyl­ammonium NH_3_ group and O1, N2, and N4 of three symmetry-related dianions (Fig. 7 ▸ *b*, Table 2 ▸). Unlike **1**, there is a strong π–π [centroid_C6/O7/N8/C9/N10_–centroid_N1–N4/C5_ distance 3.36 (2) Å, centroid_N1–N4/C5_–centroid_C6/O7/N8/C9/N10_–O7 angle, 80 (2)°] inter­action between the tetra­zolate and oxa­diazole rings. Additionally, the tetra­zolate oxygen atom does not participate in an anion–π inter­action with the oxa­diazole ring due to the stronger π–π inter­action. The oxa­diazole rings of neighboring dianions are far apart, at a centroid_C6/O7/N8/C9/N10_–centroid_C6/O7/N8/C9/N10_ distance of 4.26 (2) Å and a centroid_C6/O7/N8/C9/N10_–centroid_C6/O7/N8/C9/N10_–N10 angle of 50 (2)°, suggesting minimal π–π inter­action.

**Figure 8:**
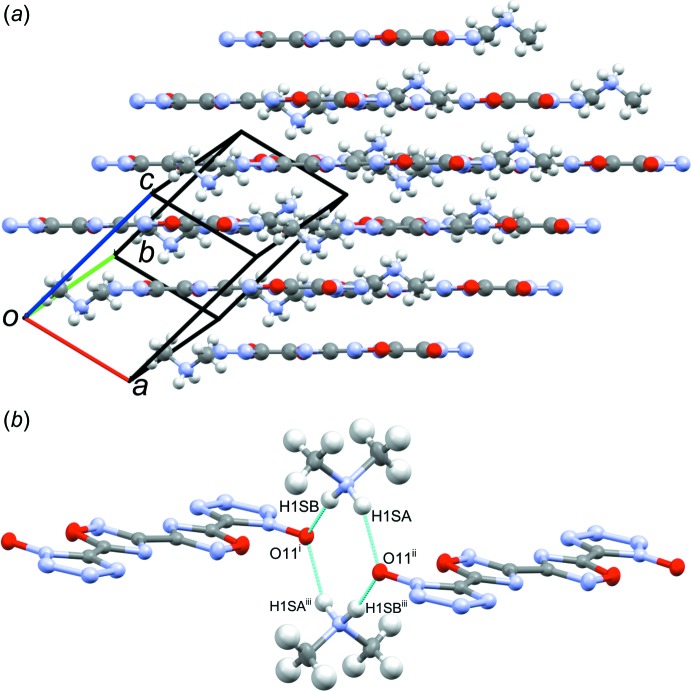
(*a*) Sheet-like packing of **3** as viewed approximately perpendicular to the (0
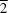
1) plane, showing the layers of dianion and associated di­methyl­ammonium cations, and (*b*) view highlighting the hydrogen-boning network (inter­molecular contacts) between di­methyl­ammonium cations and the dianions. [Symmetry codes: (i) *x*, *y*, *z* − 1; (ii) −*x* + 1, −*y*, −*z* + 1; (iii) 1 − *x*, −*y*, −*z*.]

Structure **3**, space group *P* 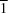, packs in a sheet-like pattern (Fig. 8 ▸ *a*), with the dianion stacked in a staggered arrangement, with the tetra­zolate ring of one dianion over the central oxa­diazole–oxa­diazole C—C bond of the dianions above and below. The oxa­diazole ring resides over the tetra­zolate–oxa­diazole C—C bond in the dianions above and below. The void space between the dianion columns is occupied by di­methyl­ammonium ions, located within the plane of the mol­ecules in an up–down arrangement. Two di­methyl­ammonium ions are positioned between the sheets, forming hydrogen bonds between the NH_2_ group and the tetra­zolate oxygen atoms of dianions in neighboring sheets (Fig. 8 ▸ *b*, Table 3 ▸). The tetra­zolate ring engages in a staggered π–π inter­action with the oxa­diazole rings of the neighboring dianion, at centroid_C6/O7/N8/C9/N10_–centroid_N1–N4/C5_ distances of 3.51 (2) and 3.99 (2) Å (the latter distance to the inversion-related oxa­diazole of the same dianion).

**Figure 9:**
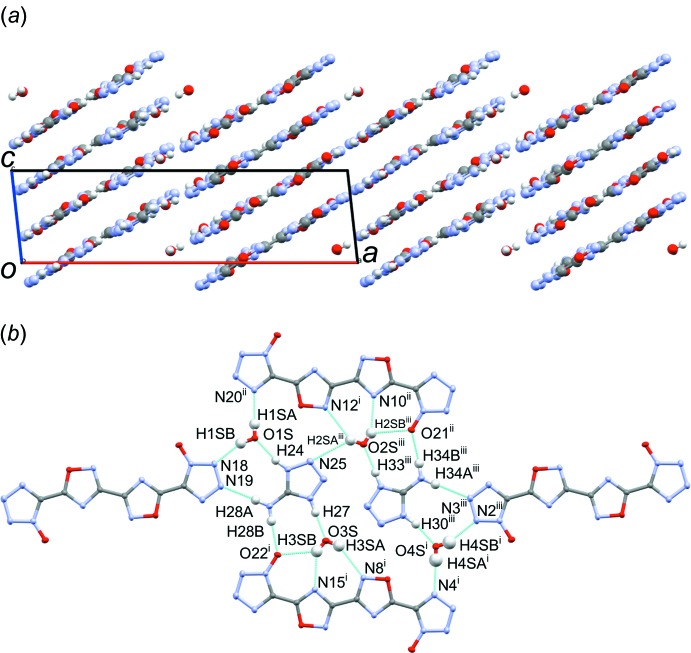
(*a*) Sheet-like packing of **4** as seen down the *b*-axis, showing the extended sheets containing both the dianion and the associated coplanar cations and solvent water, and (*b*) view highlighting the extensive in-plane hydrogen-bonding network between 5-amino­tetra­zolium, the surrounding dianions, and incorporated water mol­ecules (inter­molecular contacts). [Symmetry codes: (i) −*x*, *y* + 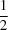, −*z* − 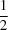; (ii) −*x*, *y* − 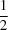, −*z* − 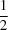; (iii) *x* − 1, *y*, *z* − 2.]

Structure **4**, space group *P*2_1_/*c*, packs in the sheet-like pattern consisting of extended sheets containing the dianion, cations, and incorporated water (Fig. 9 ▸ *a*). The 5-amino-1*H*-tetra­zol-4-ium cations and water mol­ecules surround each dianion, isolating the dianion from other dianions within the sheets. Between the sheets, the dianion only inter­acts with another dianion *via* one terminal tetra­zolate ring, with the oxygen atom of the tetra­zolate over the C—C bond between the tetra­zolate and oxa­diazole rings. Within each sheet, there is extensive hydrogen bonding between the dianions, 5-amino-1*H*-tetra­zol-4-ium, and incorporated water mol­ecules, isolating the dianions from one another in the sheet plane (Fig. 9 ▸ *b*, Table 4 ▸). The N1-tetra­zolate inter­acts with the symmetry-related N1-tetra­zolate of a neighboring mol­ecule through a π–π inter­action, with a centroid_N1–N4/C5_–centroid_N1–N4/C5_ distance of 3.69 (2) Å [N–-centroid_N1–N4/C5_–centroid_N1–N4/C5_ angle 62.0 (2)°]. The C11-oxa­diazole engages in a π–π inter­action with its symmetry equivalent as well, at a centroid_C11/N12/O13/C14/N15_–centroid_C11/N12/O13/C14/N15_ distance of 3.93 (2) Å [centroid_C11/N12/O13/C14/N15_–centroid_C11/N12/O13/C14/N15_–O13 angle 57.6 (2)°, second centroid and O13 of the same dianion]. A π–π inter­action is also seen between the N30-tetra­zolium ring and its symmetry equivalent, at a centroid_C29/N30–N33_–centroid_C29/N30–N33_ distance of 3.69 (2) Å [centroid_C29/N30–N33_–centroid_C29/N30–N33_–N31 angle 57.3 (2)°, second centroid and N31 of the same cation]. Additionally, there are two anion–π inter­actions, the first between O21 and the N1-tetra­zolate of a neighboring dianion, and the second between O21 and the C6-oxa­diazole, with an O21–centroid_N1–N4/C5_ distance of 3.33 (2) Å [O21–centroid_N1–N4/C5_–N2 angle 95.8 (2)°] and O21–centroid_C6/O7/N8/C9/N10_ 3.02 (2) Å [O21–centroid_C6/O7/N8/C9/N10_–C6 angle 76.3 (2)°].

**Figure 10:**
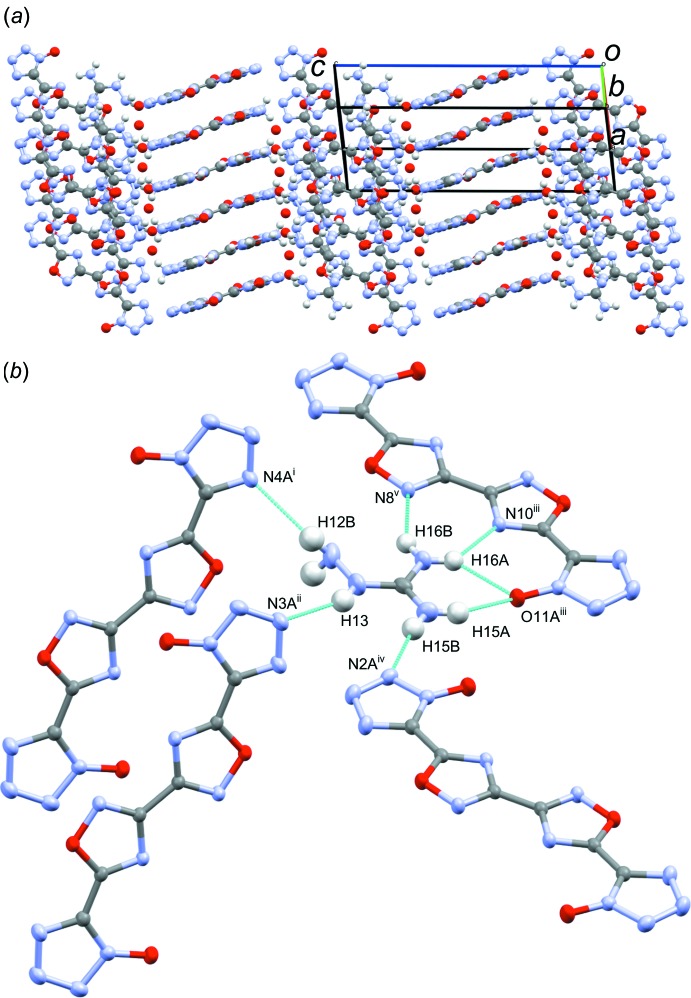
(*a*) Mixing-type packing of **5** as viewed approximately perpendicular to the (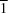
10) plane, and (*b*) view highlighting the hydrogen bonding between the dianions and amino­guanidinium cations (inter­molecular contacts, major dianion disorder component shown). [Symmetry codes: (i) −*x* + 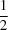, *y* + 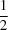, −*z* + 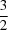; (ii) −*x* + 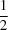, *y* − 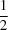, −*z* + 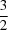; (iii) −*x* + 1, −*y* + 1, −*z* + 2; (iv) *x* − 1, *y* − 1, *z*; (v) *x*, *y* + 1, *z*.]

Structure **5**, space group *P*2_1_/*n*, packs in a mixing pattern, with columns containing stacked sheets consisting of the dianion coplanar with two amino­guanidinium cations (Fig. 10 ▸ *a*). Neighboring columns of sheets are rotated by 67° with respect to one another as a result of the hydrogen bonding of the amino group of the cation with the oxygen atom of a neighboring oxa­diazole ring. In fact, it is the hydrogen-bonding inter­action between the amino group of the amino­guanidinium cation and the oxygen atom of the oxa­diazole that directs the mixing-type packing seen in the crystal structure. The planar portion of the amino­guanidinium cation inter­acts *via* hydrogen bonds from the unsubstituted guanidinium amines to the tetra­zolate oxygen atom, oxa­diazole N8, and symmetry-related oxa­diazole N10 atoms of one dianion, and to the tetra­zolate N2 atom of a neighboring dianion (Fig. 10 ▸ *b*, Table 5 ▸). Additionally, the substituted guanidinium amine and its amine group inter­act with neighboring dianions through the tetra­zolate N3 atoms, causing the deviation from sheet-like packing. There is limited π–π inter­action between the oxa­diazole and tetra­zolate rings of neighboring dianions, with a centroid_C6/O7/N8/C9/N10_–centroid_N1*A*–N4*A*/C5*A*_ distance of 3.59 (2) Å [centroid_C6/O7/N8/C9/N10_–centroid_N1*A*–N4*A*/C5*A*_–N1*A* angle 65.4 (2)°].

As demonstrated above, it is the hydrogen-bonding networks that establish the crystal packing exhibited in each example, with π–π and anion–π inter­actions occurring if packing allows. As shown in Table 6 ▸, the densities of the crystals increase in the order **3** < **6** < **1** < **5** < **2**. Unsurprisingly, the di­methyl­ammonium, with minimal hydrogen bonding, non-inter­acting substituents, and a poor steric match for the dianion, is the least dense of the structures shown. Amino­guandinium, despite significant hydrogen bonding, exhibits a lower density as well, likely due to the directionality of the hydrogen-bond donors, which directs packing of the dianions into less efficient arrangements. Hydrazinium benefits from extensive hydrogen bonding, but the orientation of the hydrazinium directs the dianions into slightly less efficient packing than the hydroxyl­ammonium cation, preventing the staggering of the columns that allows for improved space occupation. The 5-amino-1*H*-tetra­zol-4-ium cation, with the second-highest density, packs very efficiently, in extended sheets with extensive hydrogen bonding, losing out to the hydroxyl­ammonium cation likely only due to the included water mol­ecules needed to fill in gaps between the dianions and cations. Hydroxyl­ammonium exhibits the most efficient, highest-density packing due to the directing influence and strong hydrogen-bond donating ability of the hydroxyl group, which forms a short hydrogen bond and directs the columns into a staggered arrangement, fitting the dianions slightly closer together at the point where neighboring columns meet. The range of densities, from 1.544 to 1.873 g cm^−1^, shows the significant effect that matching the hydrogen-bonding abilities and sterics of the counter-ion to the primary energetic ion has on efficient packing and, by extension, the expected performance of these ionic energetics.

## Database survey   

A search of the CSD (Version 5.38 with one update; Groom *et al.*, 2016 ▸) yields no results for structures containing 5,5′-(3,3′-bi[1,2,4-oxa­diazole]-5,5′-di­yl)bis­(1*H*-tetra­zol-1-olate). A search using 5-[3-(1,2,4-oxa­diazole)]-1*H*-tetra­zolate also yields no results. Searching for the ring fragments separately yielded 443 structures for 1,2,4-oxa­diazole and 127 structures for tetra­zol-1-olate. The closest structures to those presented herein are dimers between similar ring fragments. A search for each of the cations yields the following results: 196 structures containing hydrazinium, 99 structures containing hydroxyl­ammonium, 1,583 structures containing di­methyl­ammonium, 2,230 structures containing ammonium, 17 structures containing 5-amino-1*H*-tetra­zol-4-ium, and 130 structures containing amino­guanidinium.

## Synthesis and crystallization   

**Figure 11:**
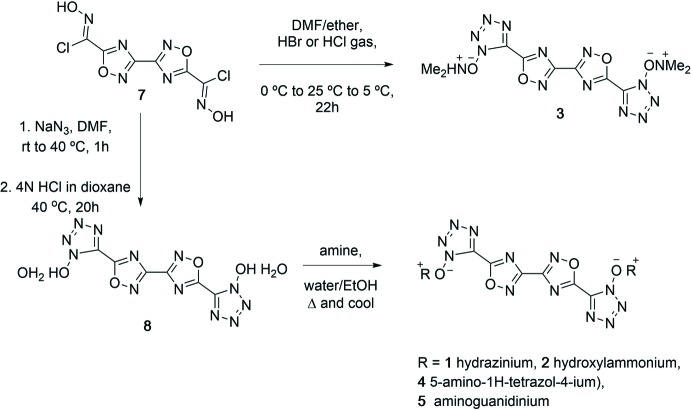
Scheme depicting synthesis pathways for the included structures.

The synthesis pathway is illustrated in Fig. 11 ▸. The synthesis and crystallization of compound **2**, and the precursors 3,3′-bis­(1,2,4-oxa­diazole)-5,5′-di­chloroxime (**6**) and 5,5′-(3,3′-bis­(1,2,4-oxa­diazole)-5,5′-di­yl)bis­(1-hy­droxy­tetra­zole) (**7**), have been described previously (Pagoria *et al.*, 2017 ▸).

Compound **1**: Dihydrate **8** (0.15 g, 0.44 mmol) was added to a 20 ml vial with water (1.5 ml) and a stir bar. Hydrazine hydrate (45 ml, 0.93 mmol) was added to the reaction mixture and heated until dissolved. Stirring was discontinued, the stir bar was removed, and the solution was allowed to cool slowly providing crystals of **1**.

Compound **3**: In a round-bottom flask, fitted with a drying tube, was suspended chloroxime **6** (967 mg, 3.3 mmol) in di­methyl­formamide (DMF) (10 ml, anhydrous), which was then cooled in an ice–water bath. Sodium azide (472 mg, 7.26 mmol) was added in portions with stirring, and the reaction was allowed to warm to room temperature. Additional DMF (10 ml) was added to the creamy mixture, and after 1.5 h, the solids went into solution. At this point, complete formation of the di­azidoxime was assumed, and cyclization to **1** proceeded as follows. A 1:1 mixture of diethyl ether/dioxane was added to the reaction mixture (100 ml total volume, anhydrous), and the solution was cooled to 273 K with an ice bath. HBr or Cl_2_ gas was bubbled into the reaction at which time the temperature increased to 298 K. Gas was added until the reaction temperature returned to approximately 278 K, and the vessel was then stoppered and allowed to stir for 22 h. The voluminous, white precipitate that formed (hygroscopic di­methyl­amonium bromide) was separated by vacuum filtration, and the filtrate was allowed to evaporate from a crystallizing dish. Upon evaporation, a white solid (**3**) in a yellow oil remained. The solid was separated from the oil by vacuum filtration (535 mg). **3** was crystallized by heating in minimal water and slow cooling.

Compound **4**: Dihydrate **7** (0.15 g, 0.44 mmol) was added to a 20 ml vial with water (1.5 ml) and a stir bar. 5-Amino­tetra­zole (0.10 g, 1.2 mmol) was added to the mixture, which was then heated with stirring until dissolved. Stirring was discontinued, the stir bar was removed, and the solution was allowed to cool slowly providing crystals of **4**.

Compound **5**: Dihydrate **7** (0.15 g, 0.44 mmol) was added to a 20 ml vial with water (1.5 ml) and a stir bar. Amino­guanidinium H_2_CO_3_ (0.24 g, 1.8 mmol) was added to the mixture, which was then heated with stirring until dissolved. During dissolution, gas evolved, the solution became clear, followed by the formation of a tan precipitate. Heating was continued until complete dissolution, followed the removal of the stir bar, and slow cooling to provide crystals of **5**.

## Refinement   

Crystal data, data collection and structure refinement details are summarized in Table 7 ▸. In **5**, the tetra­zolate ring (N1, N2, N3, N4, C5, O1) is disordered over two positons (*A* and *B*) due to a 180° rotation in the terminal tetra­zole rings. The disorder has the relative ratio of 90.7 (5):9.3 (5). CCDC deposition numbers are as follows: **1**, CCDC 1567779; **2**, CCDC 1567780; **3**, CCDC 1567783; **4**, CCDC 1567784; **5**, CCDC 1567804.

## Supplementary Material

Crystal structure: contains datablock(s) global, 1, 2, 3, 4, 5. DOI: 10.1107/S205698901800364X/pk2606sup1.cif

Structure factors: contains datablock(s) 1. DOI: 10.1107/S205698901800364X/pk26061sup9.hkl

Structure factors: contains datablock(s) 2. DOI: 10.1107/S205698901800364X/pk26062sup10.hkl

Structure factors: contains datablock(s) 3. DOI: 10.1107/S205698901800364X/pk26063sup15.hkl

Structure factors: contains datablock(s) 4. DOI: 10.1107/S205698901800364X/pk26064sup20.hkl

Structure factors: contains datablock(s) 5. DOI: 10.1107/S205698901800364X/pk26065sup8.hkl

Supporting information file. DOI: 10.1107/S205698901800364X/pk26061sup7.cml

Supporting information file. DOI: 10.1107/S205698901800364X/pk26062sup8.cml

Supporting information file. DOI: 10.1107/S205698901800364X/pk26063sup9.cml

Supporting information file. DOI: 10.1107/S205698901800364X/pk26064sup10.cml

Supporting information file. DOI: 10.1107/S205698901800364X/pk26065sup11.cml

CCDC references: 1567804, 1567784, 1567783, 1567780, 1567779

Additional supporting information:  crystallographic information; 3D view; checkCIF report

## Figures and Tables

**Figure 5:**
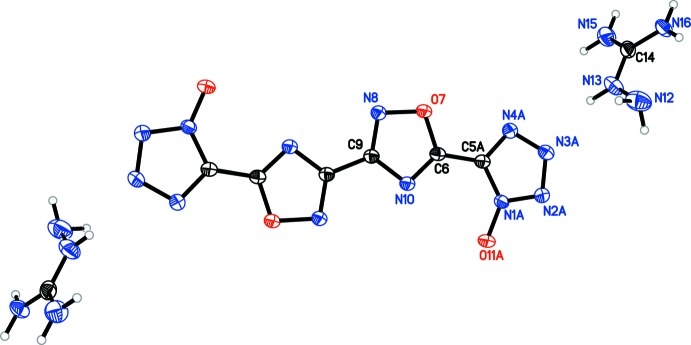
Mol­ecular structure of the major disorder component of **5**, showing the atom-labeling scheme. Displacement ellipsoids are drawn at the 50% probability level.

**Table 1:** Hydrogen-bond geometry (Å, °) for **1** [Chem scheme1]

*D*—H⋯*A*	*D*—H	H⋯*A*	*D*⋯*A*	*D*—H⋯*A*
N1*S*—H1*SA*⋯N2*S* ^i^	0.939 (17)	2.015 (18)	2.9353 (16)	166.1 (14)
N1*S*—H1*SB*⋯O1^ii^	0.904 (18)	2.007 (17)	2.7679 (15)	141.0 (14)
N1*S*—H1*SC*⋯N4^iii^	0.930 (18)	2.018 (18)	2.8778 (17)	153.0 (14)
N2*S*—H2*SA*⋯N3^iv^	0.883 (18)	2.227 (18)	3.0778 (17)	161.6 (15)
N2*S*—H2*SB*⋯O1^v^	0.882 (18)	2.071 (18)	2.8752 (15)	151.2 (15)

Symmetry codes: (i) 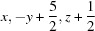; (ii) 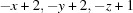; (iii) 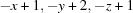; (iv) 
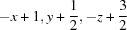
; (v) 
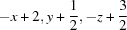
.

**Table 2:** Hydrogen-bond geometry (Å, °) for **2** [Chem scheme1]

*D*—H⋯*A*	*D*—H	H⋯*A*	*D*⋯*A*	*D*—H⋯*A*
O1*S*—H1*S*⋯O1	0.90 (2)	1.70 (2)	2.5880 (15)	169 (2)
N1*S*—H1*SA*⋯O1^i^	0.89	2.02	2.8234 (17)	149
N1*S*—H1*SB*⋯N2^ii^	0.89	2.35	2.9713 (19)	127
N1*S*—H1*SC*⋯N4^iii^	0.89	2.10	2.9425 (19)	157

Symmetry codes: (i) 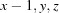; (ii) 
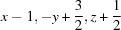
; (iii) 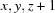.

**Table 3:** Hydrogen-bond geometry (Å, °) for **3** [Chem scheme1]

*D*—H⋯*A*	*D*—H	H⋯*A*	*D*⋯*A*	*D*—H⋯*A*
N1*S*—H1*SA*⋯O11^i^	0.91	2.01	2.8118 (14)	146
N1*S*—H1*SB*⋯O11^ii^	0.91	1.85	2.7524 (14)	169

Symmetry codes: (i) 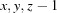; (ii) 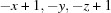.

**Table 4:** Hydrogen-bond geometry (Å, °) for **4** [Chem scheme1]

*D*—H⋯*A*	*D*—H	H⋯*A*	*D*⋯*A*	*D*—H⋯*A*
N24—H24⋯O1*S*	0.88	1.76	2.6267 (18)	169
N27—H27⋯O3*S*	0.88	1.74	2.6241 (19)	177
N28—H28*A*⋯N19	0.88	2.18	3.054 (2)	176
N28—H28*B*⋯O22^i^	0.88	1.99	2.8656 (19)	172
N30—H30⋯O4*S* ^ii^	0.88	1.75	2.605 (2)	165
N33—H33⋯O2*S*	0.88	1.78	2.6544 (19)	173
N34—H34*A*⋯N3	0.88	2.20	3.080 (2)	174
N34—H34*B*⋯O21^iii^	0.88	2.01	2.8882 (19)	175
O1*S*—H1*SA*⋯N20^iv^	0.83 (3)	1.99 (3)	2.8030 (19)	170 (2)
O1*S*—H1*SB*⋯N18	0.87 (2)	1.94 (2)	2.7758 (19)	160 (2)
O2*S*—H2*SA*⋯N12^iii^	0.81 (2)	2.39 (2)	3.0906 (18)	144 (2)
O2*S*—H2*SB*⋯N10^iii^	0.81 (3)	2.19 (2)	2.9038 (18)	148 (2)
O3*S*—H3*SA*⋯N8^i^	0.81 (2)	2.33 (2)	3.0397 (19)	147 (2)
O3*S*—H3*SB*⋯N15^i^	0.82 (3)	2.21 (3)	2.8915 (19)	141 (2)
O4*S*—H4*SA*⋯N4	0.79 (3)	2.03 (3)	2.817 (2)	177 (3)
O4*S*—H4*SB*⋯N2^iii^	0.81 (3)	2.02 (3)	2.789 (2)	157 (3)

Symmetry codes: (i) 
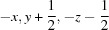
; (ii) 
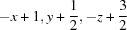
; (iii) 
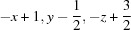
; (iv) 
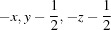
.

**Table 5:** Hydrogen-bond geometry (Å, °) for **5** [Chem scheme1]

*D*—H⋯*A*	*D*—H	H⋯*A*	*D*⋯*A*	*D*—H⋯*A*
N12—H12*B*⋯N4*A* ^i^	0.88 (1)	2.50 (1)	3.314 (4)	155 (3)
N13—H13⋯N3*A* ^ii^	0.88	2.08	2.870 (3)	149
N13—H13⋯N4*A* ^ii^	0.88	2.65	3.405 (4)	144
N15—H15*A*⋯O11*A* ^iii^	0.88	2.19	2.954 (3)	145
N15—H15*B*⋯N2*A* ^iv^	0.88	2.24	3.112 (4)	170
N16—H16*A*⋯O11*A* ^iii^	0.88	2.18	2.949 (2)	146
N16—H16*A*⋯N10^iii^	0.88	2.29	2.926 (2)	129
N16—H16*B*⋯N8^v^	0.88	2.32	3.079 (2)	145

Symmetry codes: (i) 
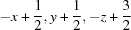
; (ii) 
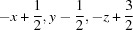
; (iii) 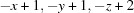; (iv) 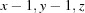; (v) 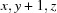.

**Table 6:** Crystal densities of each structure

**Structure ID**	**Cation**	**Density (g cm^−1^)**
**1**	hydrazinium	1.694
**2**	hydroxyl­ammonium	1.873
**3**	di­methyl­ammonium	1.544
**4**	5-amino-1*H*-tetra­zol-4-ium	1.701
**5**	amino­guanidinium	1.673

**Table:** 

	**1**	**2**	**3**
Crystal data			
Chemical formula	2N_2_H_5_^+^·C_6_N_12_O_4_^2−^	2NH_4_O^+^·C_6_N_12_O_4_^2−^	2C_2_H_8_N^+^·C_6_N_12_O_4_^2−^
*M*_r_	370.30	372.26	396.37
Crystal system, space group	Monoclinic, *P*2_1_/*c*	Monoclinic, *P*2_1_/*c*	Triclinic, *P* 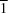
Temperature (K)	150	296	150
*a*, *b*, *c* (Å)	7.7660 (7), 13.6716 (13), 6.8655 (7)	5.1011 (9), 18.494 (3), 7.0044 (13)	6.0946 (6), 8.5197 (8), 9.2814 (9)
α, β, γ (°)	90, 95.237 (3), 90	90, 92.624 (2), 90	68.259 (3), 75.957 (3), 74.816 (3)
*V* (Å^3^)	725.89 (12)	660.1 (2)	426.28 (7)
*Z*	2	2	1
Radiation type	Mo *K*α	Mo *K*α	Mo *K*α
μ (mm^−1^)	0.14	0.17	0.12
Crystal size (mm)	0.16 × 0.15 × 0.02	0.33 × 0.19 × 0.02	0.18 × 0.12 × 0.04

Data collection			
Diffractometer	Bruker SMART APEXII CCD	Bruker SMART APEXII CCD	Bruker SMART APEXII CCD
Absorption correction	Multi-scan (*SADABS*; Bruker, 2014 ▸)	Multi-scan (*SADABS*; Bruker, 2014 ▸)	Multi-scan (*SADABS*; Bruker, 2014 ▸)
*T*_min_, *T*_max_	0.978, 0.997	0.948, 0.997	0.978, 0.995
No. of measured, independent and observed [*I* > 2σ(*I*)] reflections	6871, 1487, 1305	5834, 1358, 1152	4131, 1737, 1490
*R*_int_	0.021	0.027	0.018
(sin θ/λ)_max_ (Å^−1^)	0.628	0.628	0.625

Refinement			
*R*[*F* ^2^ > 2σ(*F* ^2^)], *wR*(*F* ^2^), *S*	0.031, 0.081, 1.04	0.034, 0.096, 1.08	0.032, 0.085, 1.04
No. of reflections	1487	1358	1737
No. of parameters	133	122	129
No. of restraints	0	0	0
H-atom treatment	Only H-atom coordinates refined	H atoms treated by a mixture of independent and constrained refinement	H-atom parameters constrained
Δρ_max_, Δρ_min_ (e Å^−3^)	0.32, −0.22	0.23, −0.26	0.27, −0.24

**Table:** 

	**4**	**5**
Crystal data		
Chemical formula	2CH_4_N_5_^+^·C_6_N_12_O_4_^2−^·4H_2_O	2CH_7_N_4_^+^·C_6_N_12_O_4_^2−^
*M*_r_	548.36	454.39
Crystal system, space group	Monoclinic, *P*2_1_/*c*	Monoclinic, *P*2_1_/*n*
Temperature (K)	150	150
*a*, *b*, *c* (Å)	24.783 (2), 12.7081 (11), 6.8396 (6)	7.9458 (4), 5.5586 (2), 20.6066 (9)
α, β, γ (°)	90, 96.289 (1), 90	90, 97.647 (2), 90
*V* (Å^3^)	2141.1 (3)	902.05 (7)
*Z*	4	2
Radiation type	Mo *K*α	Mo *K*α
μ (mm^−1^)	0.15	0.14
Crystal size (mm)	0.28 × 0.04 × 0.04	0.42 × 0.11 × 0.08

Data collection		
Diffractometer	Bruker SMART APEXII CCD	Bruker SMART APEXII CCD
Absorption correction	Multi-scan (*SADABS*; Bruker, 2014 ▸)	Multi-scan (*SADABS*; Bruker, 2014 ▸)
*T*_min_, *T*_max_	0.960, 0.994	0.944, 0.989
No. of measured, independent and observed [*I* > 2σ(*I*)] reflections	18508, 4274, 3489	7786, 1844, 1633
*R*_int_	0.027	0.020
(sin θ/λ)_max_ (Å^−1^)	0.621	0.626

Refinement		
*R*[*F* ^2^ > 2σ(*F* ^2^)], *wR*(*F* ^2^), *S*	0.036, 0.126, 1.14	0.045, 0.134, 1.06
No. of reflections	4274	1844
No. of parameters	367	206
No. of restraints	0	63
H-atom treatment	H atoms treated by a mixture of independent and constrained refinement	H atoms treated by a mixture of independent and constrained refinement
Δρ_max_, Δρ_min_ (e Å^−3^)	0.37, −0.32	0.74, −0.24

Computer programs: *APEX2* (Bruker, 2010 ▸), *SAINT* and *XPREP* (Bruker, 2014 ▸), *SHELXTL* (Sheldrick, 2008 ▸) and *SHELXL2016* (Sheldrick, 2015 ▸).

## supplementary crystallographic information

### Bis(hydrazinium) 5,5'-(3,3'-bi[1,2,4-oxadiazole]-5,5'-diyl)bis(1H-tetrazol-1-olate) (1) Crystal data

**Table:**

2N_2_H_5_^+^·C_6_N_12_O_4_^2^^−^	*F*(000) = 380
*M**_r_* = 370.30	*D*_x_ = 1.694 Mg m^−^^3^
Monoclinic, *P*2_1_/*c*	Mo *K*α radiation, λ = 0.71073 Å
*a* = 7.7660 (7) Å	Cell parameters from 2894 reflections
*b* = 13.6716 (13) Å	θ = 5.3–52.6°
*c* = 6.8655 (7) Å	µ = 0.14 mm^−^^1^
β = 95.237 (3)°	*T* = 150 K
*V* = 725.89 (12) Å^3^	Plate, colorless
*Z* = 2	0.16 × 0.15 × 0.02 mm

### Bis(hydrazinium) 5,5'-(3,3'-bi[1,2,4-oxadiazole]-5,5'-diyl)bis(1H-tetrazol-1-olate) (1) Data collection

**Table:**

Bruker SMART APEXII CCD diffractometer	1305 reflections with *I* > 2σ(*I*)
Radiation source: fine focus sealed tube	*R*_int_ = 0.021
ω scans	θ_max_ = 26.5°, θ_min_ = 2.6°
Absorption correction: multi-scan (SADABS; Bruker, 2014)	*h* = −9→9
*T*_min_ = 0.978, *T*_max_ = 0.997	*k* = −17→14
6871 measured reflections	*l* = −8→8
1487 independent reflections	

### Bis(hydrazinium) 5,5'-(3,3'-bi[1,2,4-oxadiazole]-5,5'-diyl)bis(1H-tetrazol-1-olate) (1) Refinement

**Table:**

Refinement on *F*^2^	0 restraints
Least-squares matrix: full	Hydrogen site location: difference Fourier map
*R*[*F*^2^ > 2σ(*F*^2^)] = 0.031	Only H-atom coordinates refined
*wR*(*F*^2^) = 0.081	*w* = 1/[σ^2^(*F*_o_^2^) + (0.0399*P*)^2^ + 0.3165*P*] where *P* = (*F*_o_^2^ + 2*F*_c_^2^)/3
*S* = 1.03	(Δ/σ)_max_ < 0.001
1487 reflections	Δρ_max_ = 0.32 e Å^−^^3^
133 parameters	Δρ_min_ = −0.22 e Å^−^^3^

### Bis(hydrazinium) 5,5'-(3,3'-bi[1,2,4-oxadiazole]-5,5'-diyl)bis(1H-tetrazol-1-olate) (1) Special details

**Table:**

Geometry. All esds (except the esd in the dihedral angle between two l.s. planes) are estimated using the full covariance matrix. The cell esds are taken into account individually in the estimation of esds in distances, angles and torsion angles; correlations between esds in cell parameters are only used when they are defined by crystal symmetry. An approximate (isotropic) treatment of cell esds is used for estimating esds involving l.s. planes.
Refinement. Refinement of F^2^ against ALL reflections. The weighted R-factor wR and goodness of fit S are based on F^2^, conventional R-factors R are based on F, with F set to zero for negative F^2^. The threshold expression of F^2^ > 2sigma(F^2^) is used only for calculating R-factors(gt) etc. and is not relevant to the choice of reflections for refinement. R-factors based on F^2^ are statistically about twice as large as those based on F, and R- factors based on ALL data will be even larger.

### Bis(hydrazinium) 5,5'-(3,3'-bi[1,2,4-oxadiazole]-5,5'-diyl)bis(1H-tetrazol-1-olate) (1) Fractional atomic coordinates and isotropic or equivalent isotropic displacement parameters (Å^2^)

**Table:**

	*x*	*y*	*z*	*U*_iso_*/*U*_eq_	
N1	0.91941 (14)	0.84680 (8)	0.62466 (16)	0.0184 (2)	
O1	1.08551 (12)	0.84622 (8)	0.62286 (14)	0.0241 (2)	
N2	0.83787 (15)	0.80806 (9)	0.77124 (16)	0.0235 (3)	
N3	0.67149 (15)	0.82236 (9)	0.72476 (17)	0.0255 (3)	
N4	0.64295 (15)	0.86940 (9)	0.55408 (17)	0.0226 (3)	
C5	0.79919 (17)	0.88419 (9)	0.49298 (18)	0.0180 (3)	
C6	0.83669 (16)	0.92874 (9)	0.31140 (18)	0.0180 (3)	
O7	0.70233 (12)	0.96564 (7)	0.19724 (13)	0.0226 (2)	
N8	0.77323 (15)	1.00423 (9)	0.03192 (16)	0.0221 (3)	
C9	0.93752 (17)	0.98533 (9)	0.06782 (18)	0.0177 (3)	
N10	0.98504 (14)	0.93766 (8)	0.24049 (15)	0.0179 (2)	
N1S	0.68154 (15)	1.18606 (9)	0.65235 (17)	0.0205 (3)	
H1SA	0.683 (2)	1.1856 (11)	0.789 (3)	0.025*	
H1SB	0.782 (2)	1.1628 (12)	0.614 (2)	0.025*	
H1SC	0.591 (2)	1.1482 (12)	0.596 (2)	0.025*	
N2S	0.65948 (15)	1.28374 (9)	0.57262 (16)	0.0200 (3)	
H2SA	0.560 (2)	1.3046 (12)	0.609 (2)	0.024*	
H2SB	0.740 (2)	1.3199 (12)	0.636 (2)	0.024*	

### Bis(hydrazinium) 5,5'-(3,3'-bi[1,2,4-oxadiazole]-5,5'-diyl)bis(1H-tetrazol-1-olate) (1) Atomic displacement parameters (Å^2^)

**Table:**

	*U*^11^	*U*^22^	*U*^33^	*U*^12^	*U*^13^	*U*^23^
N1	0.0201 (5)	0.0188 (6)	0.0166 (5)	−0.0012 (4)	0.0029 (4)	−0.0017 (4)
O1	0.0168 (5)	0.0353 (6)	0.0202 (5)	0.0052 (4)	0.0013 (4)	0.0017 (4)
N2	0.0282 (6)	0.0246 (6)	0.0182 (6)	−0.0028 (5)	0.0046 (5)	0.0026 (5)
N3	0.0232 (6)	0.0308 (7)	0.0230 (6)	−0.0048 (5)	0.0045 (5)	0.0024 (5)
N4	0.0212 (6)	0.0251 (6)	0.0217 (6)	−0.0040 (5)	0.0033 (4)	0.0012 (5)
C5	0.0194 (6)	0.0159 (6)	0.0186 (6)	−0.0012 (5)	0.0014 (5)	−0.0015 (5)
C6	0.0186 (6)	0.0159 (6)	0.0191 (6)	−0.0001 (5)	−0.0007 (5)	−0.0009 (5)
O7	0.0184 (5)	0.0280 (5)	0.0215 (5)	0.0003 (4)	0.0016 (4)	0.0060 (4)
N8	0.0228 (6)	0.0236 (6)	0.0200 (6)	−0.0005 (5)	0.0021 (4)	0.0049 (5)
C9	0.0208 (6)	0.0142 (6)	0.0178 (6)	0.0002 (5)	−0.0001 (5)	0.0005 (5)
N10	0.0196 (5)	0.0169 (5)	0.0172 (5)	0.0004 (4)	0.0016 (4)	0.0014 (4)
N1S	0.0172 (6)	0.0265 (6)	0.0177 (6)	−0.0001 (5)	0.0013 (4)	−0.0002 (5)
N2S	0.0178 (6)	0.0233 (6)	0.0188 (5)	−0.0007 (5)	0.0015 (5)	−0.0025 (5)

### Bis(hydrazinium) 5,5'-(3,3'-bi[1,2,4-oxadiazole]-5,5'-diyl)bis(1H-tetrazol-1-olate) (1) Geometric parameters (Å, º)

**Table:**

N1—O1	1.2912 (14)	N8—C9	1.3031 (18)
N1—C5	1.3402 (17)	C9—N10	1.3735 (16)
N1—N2	1.3455 (16)	C9—C9^i^	1.461 (3)
N2—N3	1.3170 (17)	N1S—N2S	1.4476 (16)
N3—N4	1.3373 (17)	N1S—H1SA	0.939 (17)
N4—C5	1.3349 (17)	N1S—H1SB	0.904 (18)
C5—C6	1.4409 (18)	N1S—H1SC	0.930 (18)
C6—N10	1.2968 (17)	N2S—H2SA	0.883 (18)
C6—O7	1.3447 (16)	N2S—H2SB	0.882 (18)
O7—N8	1.4085 (14)		
			
O1—N1—C5	129.12 (11)	N8—C9—N10	116.02 (12)
O1—N1—N2	122.92 (11)	N8—C9—C9^i^	121.39 (14)
C5—N1—N2	107.96 (11)	N10—C9—C9^i^	122.59 (15)
N3—N2—N1	106.18 (11)	C6—N10—C9	100.99 (11)
N2—N3—N4	111.36 (11)	N2S—N1S—H1SA	112.1 (10)
C5—N4—N3	105.40 (11)	N2S—N1S—H1SB	106.9 (11)
N4—C5—N1	109.11 (11)	H1SA—N1S—H1SB	110.7 (15)
N4—C5—C6	126.67 (12)	N2S—N1S—H1SC	107.3 (10)
N1—C5—C6	124.16 (12)	H1SA—N1S—H1SC	110.6 (14)
N10—C6—O7	114.49 (11)	H1SB—N1S—H1SC	109.1 (14)
N10—C6—C5	128.41 (12)	N1S—N2S—H2SA	105.6 (11)
O7—C6—C5	117.10 (11)	N1S—N2S—H2SB	106.0 (11)
C6—O7—N8	105.70 (10)	H2SA—N2S—H2SB	106.4 (15)
C9—N8—O7	102.79 (10)		
			
O1—N1—N2—N3	179.57 (11)	N4—C5—C6—O7	5.1 (2)
C5—N1—N2—N3	0.31 (14)	N1—C5—C6—O7	−177.99 (12)
N1—N2—N3—N4	−0.35 (15)	N10—C6—O7—N8	−0.61 (15)
N2—N3—N4—C5	0.26 (15)	C5—C6—O7—N8	179.95 (11)
N3—N4—C5—N1	−0.06 (15)	C6—O7—N8—C9	0.25 (13)
N3—N4—C5—C6	177.23 (13)	O7—N8—C9—N10	0.14 (15)
O1—N1—C5—N4	−179.36 (12)	O7—N8—C9—C9^i^	−179.12 (15)
N2—N1—C5—N4	−0.16 (15)	O7—C6—N10—C9	0.65 (14)
O1—N1—C5—C6	3.3 (2)	C5—C6—N10—C9	−179.98 (13)
N2—N1—C5—C6	−177.52 (12)	N8—C9—N10—C6	−0.48 (15)
N4—C5—C6—N10	−174.25 (13)	C9^i^—C9—N10—C6	178.77 (15)
N1—C5—C6—N10	2.6 (2)		

Symmetry code: (i) −*x*+2, −*y*+2, −*z*.

### Bis(hydrazinium) 5,5'-(3,3'-bi[1,2,4-oxadiazole]-5,5'-diyl)bis(1H-tetrazol-1-olate) (1) Hydrogen-bond geometry (Å, º)

**Table:**

*D*—H···*A*	*D*—H	H···*A*	*D*···*A*	*D*—H···*A*
N1*S*—H1*SA*···N2*S*^ii^	0.939 (17)	2.015 (18)	2.9353 (16)	166.1 (14)
N1*S*—H1*SB*···O1^iii^	0.904 (18)	2.007 (17)	2.7679 (15)	141.0 (14)
N1*S*—H1*SC*···N4^iv^	0.930 (18)	2.018 (18)	2.8778 (17)	153.0 (14)
N2*S*—H2*SA*···N3^v^	0.883 (18)	2.227 (18)	3.0778 (17)	161.6 (15)
N2*S*—H2*SB*···O1^vi^	0.882 (18)	2.071 (18)	2.8752 (15)	151.2 (15)

Symmetry codes: (ii) *x*, −*y*+5/2, *z*+1/2; (iii) −*x*+2, −*y*+2, −*z*+1; (iv) −*x*+1, −*y*+2, −*z*+1; (v) −*x*+1, *y*+1/2, −*z*+3/2; (vi) −*x*+2, *y*+1/2, −*z*+3/2.

### Bis(hydroxyammonium) 5,5'-(3,3'-bi[1,2,4-oxadiazole]-5,5'-diyl)bis(1H-tetrazol-1-olate) (2) Crystal data

**Table:**

2NH_4_O^+^·C_6_N_12_O_4_^2^^−^	*F*(000) = 380
*M**_r_* = 372.26	*D*_x_ = 1.873 Mg m^−^^3^
Monoclinic, *P*2_1_/*c*	Mo *K*α radiation, λ = 0.71073 Å
*a* = 5.1011 (9) Å	Cell parameters from 2126 reflections
*b* = 18.494 (3) Å	θ = 4.4–51.8°
*c* = 7.0044 (13) Å	µ = 0.17 mm^−^^1^
β = 92.624 (2)°	*T* = 296 K
*V* = 660.1 (2) Å^3^	Plate, colorless
*Z* = 2	0.33 × 0.19 × 0.02 mm

### Bis(hydroxyammonium) 5,5'-(3,3'-bi[1,2,4-oxadiazole]-5,5'-diyl)bis(1H-tetrazol-1-olate) (2) Data collection

**Table:**

Bruker SMART APEXII CCD diffractometer	1152 reflections with *I* > 2σ(*I*)
Radiation source: fine focus sealed tube	*R*_int_ = 0.027
ω scans	θ_max_ = 26.5°, θ_min_ = 2.2°
Absorption correction: multi-scan (SADABS; Bruker, 2014)	*h* = −6→6
*T*_min_ = 0.948, *T*_max_ = 0.997	*k* = −23→23
5834 measured reflections	*l* = −8→8
1358 independent reflections	

### Bis(hydroxyammonium) 5,5'-(3,3'-bi[1,2,4-oxadiazole]-5,5'-diyl)bis(1H-tetrazol-1-olate) (2) Refinement

**Table:**

Refinement on *F*^2^	0 restraints
Least-squares matrix: full	Hydrogen site location: mixed
*R*[*F*^2^ > 2σ(*F*^2^)] = 0.034	H atoms treated by a mixture of independent and constrained refinement
*wR*(*F*^2^) = 0.096	*w* = 1/[σ^2^(*F*_o_^2^) + (0.0582*P*)^2^ + 0.1422*P*] where *P* = (*F*_o_^2^ + 2*F*_c_^2^)/3
*S* = 1.08	(Δ/σ)_max_ < 0.001
1358 reflections	Δρ_max_ = 0.23 e Å^−^^3^
122 parameters	Δρ_min_ = −0.25 e Å^−^^3^

### Bis(hydroxyammonium) 5,5'-(3,3'-bi[1,2,4-oxadiazole]-5,5'-diyl)bis(1H-tetrazol-1-olate) (2) Special details

**Table:**

Geometry. All esds (except the esd in the dihedral angle between two l.s. planes) are estimated using the full covariance matrix. The cell esds are taken into account individually in the estimation of esds in distances, angles and torsion angles; correlations between esds in cell parameters are only used when they are defined by crystal symmetry. An approximate (isotropic) treatment of cell esds is used for estimating esds involving l.s. planes.
Refinement. Refinement of F^2^ against ALL reflections. The weighted R-factor wR and goodness of fit S are based on F^2^, conventional R-factors R are based on F, with F set to zero for negative F^2^. The threshold expression of F^2^ > 2sigma(F^2^) is used only for calculating R-factors(gt) etc. and is not relevant to the choice of reflections for refinement. R-factors based on F^2^ are statistically about twice as large as those based on F, and R- factors based on ALL data will be even larger.

### Bis(hydroxyammonium) 5,5'-(3,3'-bi[1,2,4-oxadiazole]-5,5'-diyl)bis(1H-tetrazol-1-olate) (2) Fractional atomic coordinates and isotropic or equivalent isotropic displacement parameters (Å^2^)

**Table:**

	*x*	*y*	*z*	*U*_iso_*/*U*_eq_	
O1	0.26439 (19)	0.64777 (6)	0.49548 (14)	0.0199 (3)	
N1	0.2362 (2)	0.64703 (6)	0.30834 (18)	0.0170 (3)	
N2	0.3978 (2)	0.68219 (7)	0.19563 (19)	0.0208 (3)	
N3	0.3121 (3)	0.66943 (7)	0.01809 (19)	0.0227 (3)	
N4	0.0995 (2)	0.62648 (7)	0.01466 (18)	0.0204 (3)	
C5	0.0547 (3)	0.61257 (8)	0.1968 (2)	0.0168 (3)	
C6	−0.1502 (3)	0.56752 (8)	0.2694 (2)	0.0166 (3)	
O7	−0.2867 (2)	0.52656 (6)	0.14236 (15)	0.0237 (3)	
N8	−0.4729 (3)	0.48950 (7)	0.24763 (19)	0.0234 (3)	
C9	−0.4235 (3)	0.51234 (8)	0.4207 (2)	0.0164 (3)	
N10	−0.2225 (2)	0.56162 (7)	0.44333 (18)	0.0174 (3)	
O1S	−0.1197 (2)	0.72437 (6)	0.61500 (17)	0.0248 (3)	
H1S	0.003 (4)	0.6930 (12)	0.578 (3)	0.037*	
N1S	−0.2862 (2)	0.68301 (7)	0.72712 (19)	0.0187 (3)	
H1SA	−0.392760	0.656491	0.651209	0.028*	
H1SB	−0.381130	0.712375	0.797232	0.028*	
H1SC	−0.190031	0.654038	0.803773	0.028*	

### Bis(hydroxyammonium) 5,5'-(3,3'-bi[1,2,4-oxadiazole]-5,5'-diyl)bis(1H-tetrazol-1-olate) (2) Atomic displacement parameters (Å^2^)

**Table:**

	*U*^11^	*U*^22^	*U*^33^	*U*^12^	*U*^13^	*U*^23^
O1	0.0150 (5)	0.0290 (6)	0.0157 (6)	0.0000 (4)	0.0006 (4)	−0.0016 (4)
N1	0.0127 (6)	0.0200 (6)	0.0185 (7)	0.0003 (5)	0.0021 (5)	0.0014 (5)
N2	0.0149 (6)	0.0237 (7)	0.0242 (7)	−0.0007 (5)	0.0056 (5)	0.0018 (5)
N3	0.0181 (6)	0.0263 (7)	0.0241 (7)	0.0005 (5)	0.0056 (5)	0.0034 (5)
N4	0.0168 (6)	0.0243 (7)	0.0205 (7)	0.0009 (5)	0.0040 (5)	0.0027 (5)
C5	0.0128 (6)	0.0198 (7)	0.0178 (8)	0.0022 (5)	0.0003 (5)	0.0005 (5)
C6	0.0135 (7)	0.0182 (7)	0.0180 (8)	0.0013 (5)	−0.0004 (6)	0.0001 (6)
O7	0.0235 (6)	0.0295 (6)	0.0181 (6)	−0.0096 (5)	0.0026 (5)	−0.0011 (4)
N8	0.0219 (7)	0.0272 (7)	0.0213 (7)	−0.0092 (5)	0.0036 (6)	0.0010 (5)
C9	0.0134 (7)	0.0162 (7)	0.0196 (8)	0.0007 (5)	0.0005 (6)	0.0010 (6)
N10	0.0134 (6)	0.0198 (6)	0.0192 (7)	−0.0013 (5)	0.0014 (5)	0.0008 (5)
O1S	0.0181 (5)	0.0238 (6)	0.0335 (7)	−0.0016 (5)	0.0127 (5)	0.0016 (5)
N1S	0.0140 (6)	0.0226 (7)	0.0199 (7)	−0.0015 (5)	0.0032 (5)	0.0008 (5)

### Bis(hydroxyammonium) 5,5'-(3,3'-bi[1,2,4-oxadiazole]-5,5'-diyl)bis(1H-tetrazol-1-olate) (2) Geometric parameters (Å, º)

**Table:**

O1—N1	1.3122 (16)	O7—N8	1.4071 (16)
N1—N2	1.3361 (18)	N8—C9	1.298 (2)
N1—C5	1.3443 (19)	C9—N10	1.3755 (19)
N2—N3	1.3204 (19)	C9—C9^i^	1.459 (3)
N3—N4	1.3436 (18)	O1S—N1S	1.4087 (16)
N4—C5	1.332 (2)	O1S—H1S	0.90 (2)
C5—C6	1.447 (2)	N1S—H1SA	0.8900
C6—N10	1.294 (2)	N1S—H1SB	0.8900
C6—O7	1.3391 (18)	N1S—H1SC	0.8900
			
O1—N1—N2	122.87 (12)	C9—N8—O7	102.96 (11)
O1—N1—C5	128.79 (12)	N8—C9—N10	115.78 (13)
N2—N1—C5	108.33 (12)	N8—C9—C9^i^	121.42 (17)
N3—N2—N1	106.43 (12)	N10—C9—C9^i^	122.79 (17)
N2—N3—N4	110.78 (12)	C6—N10—C9	100.99 (12)
C5—N4—N3	105.72 (13)	N1S—O1S—H1S	104.8 (13)
N4—C5—N1	108.74 (13)	O1S—N1S—H1SA	109.5
N4—C5—C6	127.32 (13)	O1S—N1S—H1SB	109.5
N1—C5—C6	123.94 (13)	H1SA—N1S—H1SB	109.5
N10—C6—O7	114.58 (13)	O1S—N1S—H1SC	109.5
N10—C6—C5	128.39 (14)	H1SA—N1S—H1SC	109.5
O7—C6—C5	117.03 (13)	H1SB—N1S—H1SC	109.5
C6—O7—N8	105.68 (11)		
			
O1—N1—N2—N3	179.78 (12)	N4—C5—C6—O7	10.8 (2)
C5—N1—N2—N3	0.58 (15)	N1—C5—C6—O7	−168.32 (13)
N1—N2—N3—N4	−0.30 (15)	N10—C6—O7—N8	0.34 (16)
N2—N3—N4—C5	−0.10 (16)	C5—C6—O7—N8	−179.17 (12)
N3—N4—C5—N1	0.46 (15)	C6—O7—N8—C9	−0.35 (15)
N3—N4—C5—C6	−178.77 (14)	O7—N8—C9—N10	0.29 (17)
O1—N1—C5—N4	−179.80 (13)	O7—N8—C9—C9^i^	179.29 (16)
N2—N1—C5—N4	−0.66 (16)	O7—C6—N10—C9	−0.17 (16)
O1—N1—C5—C6	−0.5 (2)	C5—C6—N10—C9	179.28 (14)
N2—N1—C5—C6	178.61 (13)	N8—C9—N10—C6	−0.09 (17)
N4—C5—C6—N10	−168.63 (15)	C9^i^—C9—N10—C6	−179.08 (17)
N1—C5—C6—N10	12.2 (2)		

Symmetry code: (i) −*x*−1, −*y*+1, −*z*+1.

### Bis(hydroxyammonium) 5,5'-(3,3'-bi[1,2,4-oxadiazole]-5,5'-diyl)bis(1H-tetrazol-1-olate) (2) Hydrogen-bond geometry (Å, º)

**Table:**

*D*—H···*A*	*D*—H	H···*A*	*D*···*A*	*D*—H···*A*
O1*S*—H1*S*···O1	0.90 (2)	1.70 (2)	2.5880 (15)	169 (2)
N1*S*—H1*SA*···O1^ii^	0.89	2.02	2.8234 (17)	149
N1*S*—H1*SB*···N2^iii^	0.89	2.35	2.9713 (19)	127
N1*S*—H1*SC*···N4^iv^	0.89	2.10	2.9425 (19)	157

Symmetry codes: (ii) *x*−1, *y*, *z*; (iii) *x*−1, −*y*+3/2, *z*+1/2; (iv) *x*, *y*, *z*+1.

### Dimethylammonium 5,5'-(3,3'-bi[1,2,4-oxadiazole]-5,5'-diyl)bis(1H-tetrazol-1-olate) (3) Crystal data

**Table:**

2C_2_H_8_N^+^·C_6_N_12_O_4_^2^^−^	*Z* = 1
*M**_r_* = 396.37	*F*(000) = 206
Triclinic, *P*1	*D*_x_ = 1.544 Mg m^−^^3^
*a* = 6.0946 (6) Å	Mo *K*α radiation, λ = 0.71073 Å
*b* = 8.5197 (8) Å	Cell parameters from 1925 reflections
*c* = 9.2814 (9) Å	θ = 4.8–52.6°
α = 68.259 (3)°	µ = 0.12 mm^−^^1^
β = 75.957 (3)°	*T* = 150 K
γ = 74.816 (3)°	Plate, yellow
*V* = 426.28 (7) Å^3^	0.18 × 0.12 × 0.04 mm

### Dimethylammonium 5,5'-(3,3'-bi[1,2,4-oxadiazole]-5,5'-diyl)bis(1H-tetrazol-1-olate) (3) Data collection

**Table:**

Bruker SMART APEXII CCD diffractometer	1490 reflections with *I* > 2σ(*I*)
Radiation source: fine focus sealed tube	*R*_int_ = 0.018
ω scans	θ_max_ = 26.4°, θ_min_ = 2.4°
Absorption correction: multi-scan (SADABS; Bruker, 2014)	*h* = −7→7
*T*_min_ = 0.978, *T*_max_ = 0.995	*k* = −10→9
4131 measured reflections	*l* = −11→11
1737 independent reflections	

### Dimethylammonium 5,5'-(3,3'-bi[1,2,4-oxadiazole]-5,5'-diyl)bis(1H-tetrazol-1-olate) (3) Refinement

**Table:**

Refinement on *F*^2^	0 restraints
Least-squares matrix: full	Hydrogen site location: inferred from neighbouring sites
*R*[*F*^2^ > 2σ(*F*^2^)] = 0.032	H-atom parameters constrained
*wR*(*F*^2^) = 0.085	*w* = 1/[σ^2^(*F*_o_^2^) + (0.0432*P*)^2^ + 0.1053*P*] where *P* = (*F*_o_^2^ + 2*F*_c_^2^)/3
*S* = 1.04	(Δ/σ)_max_ < 0.001
1737 reflections	Δρ_max_ = 0.27 e Å^−^^3^
129 parameters	Δρ_min_ = −0.23 e Å^−^^3^

### Dimethylammonium 5,5'-(3,3'-bi[1,2,4-oxadiazole]-5,5'-diyl)bis(1H-tetrazol-1-olate) (3) Special details

**Table:**

Geometry. All esds (except the esd in the dihedral angle between two l.s. planes) are estimated using the full covariance matrix. The cell esds are taken into account individually in the estimation of esds in distances, angles and torsion angles; correlations between esds in cell parameters are only used when they are defined by crystal symmetry. An approximate (isotropic) treatment of cell esds is used for estimating esds involving l.s. planes.
Refinement. Refinement of F^2^ against ALL reflections. The weighted R-factor wR and goodness of fit S are based on F^2^, conventional R-factors R are based on F, with F set to zero for negative F^2^. The threshold expression of F^2^ > 2sigma(F^2^) is used only for calculating R-factors(gt) etc. and is not relevant to the choice of reflections for refinement. R-factors based on F^2^ are statistically about twice as large as those based on F, and R- factors based on ALL data will be even larger.

### Dimethylammonium 5,5'-(3,3'-bi[1,2,4-oxadiazole]-5,5'-diyl)bis(1H-tetrazol-1-olate) (3) Fractional atomic coordinates and isotropic or equivalent isotropic displacement parameters (Å^2^)

**Table:**

	*x*	*y*	*z*	*U*_iso_*/*U*_eq_	
N1	0.59547 (17)	0.36241 (13)	0.87451 (12)	0.0211 (2)	
N2	0.73348 (18)	0.44045 (14)	0.90588 (12)	0.0246 (3)	
N3	0.69908 (19)	0.60180 (14)	0.81186 (13)	0.0259 (3)	
N4	0.54333 (18)	0.62953 (14)	0.72093 (12)	0.0241 (2)	
C5	0.4791 (2)	0.47875 (15)	0.76160 (14)	0.0205 (3)	
C6	0.3133 (2)	0.44918 (15)	0.69185 (14)	0.0201 (3)	
O7	0.25452 (15)	0.29342 (11)	0.74431 (10)	0.0248 (2)	
N8	0.09343 (19)	0.30587 (14)	0.65217 (13)	0.0249 (3)	
C9	0.0765 (2)	0.46521 (15)	0.55884 (13)	0.0201 (3)	
N10	0.20941 (17)	0.56041 (13)	0.57819 (11)	0.0210 (2)	
O11	0.58447 (16)	0.20008 (11)	0.94750 (10)	0.0258 (2)	
N1S	0.26051 (19)	0.04614 (13)	0.19850 (12)	0.0249 (3)	
H1SA	0.344680	0.128501	0.137883	0.030*	
H1SB	0.312665	−0.045306	0.162814	0.030*	
C2S	0.0164 (3)	0.11493 (19)	0.1796 (2)	0.0399 (4)	
H2SA	−0.075472	0.026667	0.243664	0.060*	
H2SB	0.002362	0.147390	0.068857	0.060*	
H2SC	−0.039593	0.216384	0.213810	0.060*	
C3S	0.2991 (3)	−0.00899 (18)	0.36271 (16)	0.0326 (3)	
H3SA	0.232927	0.086212	0.404837	0.049*	
H3SB	0.464886	−0.042153	0.365781	0.049*	
H3SC	0.224951	−0.107586	0.426403	0.049*	

### Dimethylammonium 5,5'-(3,3'-bi[1,2,4-oxadiazole]-5,5'-diyl)bis(1H-tetrazol-1-olate) (3) Atomic displacement parameters (Å^2^)

**Table:**

	*U*^11^	*U*^22^	*U*^33^	*U*^12^	*U*^13^	*U*^23^
N1	0.0216 (5)	0.0208 (5)	0.0207 (5)	−0.0039 (4)	−0.0006 (4)	−0.0083 (4)
N2	0.0227 (5)	0.0291 (6)	0.0260 (5)	−0.0061 (4)	−0.0025 (4)	−0.0136 (5)
N3	0.0264 (6)	0.0266 (6)	0.0270 (6)	−0.0078 (4)	−0.0014 (4)	−0.0116 (5)
N4	0.0258 (5)	0.0245 (5)	0.0246 (5)	−0.0083 (4)	−0.0018 (4)	−0.0100 (4)
C5	0.0214 (6)	0.0208 (6)	0.0197 (6)	−0.0042 (5)	−0.0007 (5)	−0.0084 (5)
C6	0.0211 (6)	0.0187 (6)	0.0205 (6)	−0.0047 (5)	0.0013 (5)	−0.0086 (5)
O7	0.0293 (5)	0.0193 (4)	0.0275 (5)	−0.0079 (4)	−0.0099 (4)	−0.0039 (4)
N8	0.0283 (6)	0.0232 (5)	0.0263 (5)	−0.0073 (4)	−0.0093 (4)	−0.0068 (4)
C9	0.0206 (6)	0.0208 (6)	0.0199 (6)	−0.0045 (5)	0.0002 (5)	−0.0096 (5)
N10	0.0224 (5)	0.0199 (5)	0.0219 (5)	−0.0056 (4)	−0.0027 (4)	−0.0077 (4)
O11	0.0326 (5)	0.0183 (4)	0.0250 (5)	−0.0044 (4)	−0.0041 (4)	−0.0061 (3)
N1S	0.0301 (6)	0.0178 (5)	0.0254 (5)	−0.0062 (4)	−0.0009 (4)	−0.0068 (4)
C2S	0.0344 (8)	0.0275 (7)	0.0587 (10)	−0.0035 (6)	−0.0172 (7)	−0.0110 (7)
C3S	0.0452 (8)	0.0251 (7)	0.0269 (7)	−0.0057 (6)	−0.0071 (6)	−0.0077 (5)

### Dimethylammonium 5,5'-(3,3'-bi[1,2,4-oxadiazole]-5,5'-diyl)bis(1H-tetrazol-1-olate) (3) Geometric parameters (Å, º)

**Table:**

N1—O11	1.3071 (13)	C9—C9^i^	1.463 (2)
N1—N2	1.3377 (15)	N1S—C2S	1.4767 (18)
N1—C5	1.3440 (16)	N1S—C3S	1.4780 (17)
N2—N3	1.3207 (15)	N1S—H1SA	0.9100
N3—N4	1.3390 (16)	N1S—H1SB	0.9100
N4—C5	1.3334 (16)	C2S—H2SA	0.9800
C5—C6	1.4410 (18)	C2S—H2SB	0.9800
C6—N10	1.2964 (16)	C2S—H2SC	0.9800
C6—O7	1.3460 (14)	C3S—H3SA	0.9800
O7—N8	1.4115 (14)	C3S—H3SB	0.9800
N8—C9	1.3049 (16)	C3S—H3SC	0.9800
C9—N10	1.3671 (16)		
			
O11—N1—N2	122.67 (10)	C2S—N1S—C3S	113.54 (12)
O11—N1—C5	129.09 (11)	C2S—N1S—H1SA	108.9
N2—N1—C5	108.24 (10)	C3S—N1S—H1SA	108.9
N3—N2—N1	106.11 (10)	C2S—N1S—H1SB	108.9
N2—N3—N4	111.33 (10)	C3S—N1S—H1SB	108.9
C5—N4—N3	105.34 (10)	H1SA—N1S—H1SB	107.7
N4—C5—N1	108.98 (11)	N1S—C2S—H2SA	109.5
N4—C5—C6	124.29 (11)	N1S—C2S—H2SB	109.5
N1—C5—C6	126.73 (12)	H2SA—C2S—H2SB	109.5
N10—C6—O7	113.91 (11)	N1S—C2S—H2SC	109.5
N10—C6—C5	126.23 (11)	H2SA—C2S—H2SC	109.5
O7—C6—C5	119.86 (11)	H2SB—C2S—H2SC	109.5
C6—O7—N8	105.99 (9)	N1S—C3S—H3SA	109.5
C9—N8—O7	102.47 (10)	N1S—C3S—H3SB	109.5
N8—C9—N10	116.05 (11)	H3SA—C3S—H3SB	109.5
N8—C9—C9^i^	121.02 (14)	N1S—C3S—H3SC	109.5
N10—C9—C9^i^	122.94 (13)	H3SA—C3S—H3SC	109.5
C6—N10—C9	101.58 (10)	H3SB—C3S—H3SC	109.5
			
O11—N1—N2—N3	−179.65 (9)	N4—C5—C6—O7	−177.99 (10)
C5—N1—N2—N3	−0.10 (13)	N1—C5—C6—O7	2.86 (18)
N1—N2—N3—N4	−0.09 (13)	N10—C6—O7—N8	0.34 (13)
N2—N3—N4—C5	0.25 (13)	C5—C6—O7—N8	−179.34 (10)
N3—N4—C5—N1	−0.31 (13)	C6—O7—N8—C9	−0.33 (12)
N3—N4—C5—C6	−179.59 (11)	O7—N8—C9—N10	0.24 (13)
O11—N1—C5—N4	179.77 (10)	O7—N8—C9—C9^i^	−179.89 (13)
N2—N1—C5—N4	0.26 (13)	O7—C6—N10—C9	−0.18 (13)
O11—N1—C5—C6	−1.0 (2)	C5—C6—N10—C9	179.47 (11)
N2—N1—C5—C6	179.52 (11)	N8—C9—N10—C6	−0.05 (14)
N4—C5—C6—N10	2.38 (19)	C9^i^—C9—N10—C6	−179.91 (14)
N1—C5—C6—N10	−176.77 (11)		

Symmetry code: (i) −*x*, −*y*+1, −*z*+1.

### Dimethylammonium 5,5'-(3,3'-bi[1,2,4-oxadiazole]-5,5'-diyl)bis(1H-tetrazol-1-olate) (3) Hydrogen-bond geometry (Å, º)

**Table:**

*D*—H···*A*	*D*—H	H···*A*	*D*···*A*	*D*—H···*A*
N1*S*—H1*SA*···O11^ii^	0.91	2.01	2.8118 (14)	146
N1*S*—H1*SB*···O11^iii^	0.91	1.85	2.7524 (14)	169

Symmetry codes: (ii) *x*, *y*, *z*−1; (iii) −*x*+1, −*y*, −*z*+1.

### Bis(5-amino-1H-tetrazol-4-ium) 5,5'-(3,3'-bi[1,2,4-oxadiazole]-5,5'-diyl)bis(1H-tetrazol-1-olate) (4) Crystal data

**Table:**

2CH_4_N_5_^+^·C_6_N_12_O_4_^2^^−^·4H_2_O	*F*(000) = 1128
*M**_r_* = 548.36	*D*_x_ = 1.701 Mg m^−^^3^
Monoclinic, *P*2_1_/*c*	Mo *K*α radiation, λ = 0.71073 Å
*a* = 24.783 (2) Å	Cell parameters from 5868 reflections
*b* = 12.7081 (11) Å	θ = 5.9–52.2°
*c* = 6.8396 (6) Å	µ = 0.15 mm^−^^1^
β = 96.289 (1)°	*T* = 150 K
*V* = 2141.1 (3) Å^3^	Rod, colorless
*Z* = 4	0.28 × 0.04 × 0.04 mm

### Bis(5-amino-1H-tetrazol-4-ium) 5,5'-(3,3'-bi[1,2,4-oxadiazole]-5,5'-diyl)bis(1H-tetrazol-1-olate) (4) Data collection

**Table:**

Bruker SMART APEXII CCD diffractometer	3489 reflections with *I* > 2σ(*I*)
Radiation source: fine focus sealed tube	*R*_int_ = 0.027
ω scans	θ_max_ = 26.2°, θ_min_ = 0.8°
Absorption correction: multi-scan (SADABS; Bruker, 2014)	*h* = −30→29
*T*_min_ = 0.960, *T*_max_ = 0.994	*k* = −15→15
18508 measured reflections	*l* = −8→8
4274 independent reflections	

### Bis(5-amino-1H-tetrazol-4-ium) 5,5'-(3,3'-bi[1,2,4-oxadiazole]-5,5'-diyl)bis(1H-tetrazol-1-olate) (4) Refinement

**Table:**

Refinement on *F*^2^	0 restraints
Least-squares matrix: full	Hydrogen site location: mixed
*R*[*F*^2^ > 2σ(*F*^2^)] = 0.036	H atoms treated by a mixture of independent and constrained refinement
*wR*(*F*^2^) = 0.126	*w* = 1/[σ^2^(*F*_o_^2^) + (0.0807*P*)^2^ + 0.1085*P*] where *P* = (*F*_o_^2^ + 2*F*_c_^2^)/3
*S* = 1.14	(Δ/σ)_max_ < 0.001
4274 reflections	Δρ_max_ = 0.37 e Å^−^^3^
367 parameters	Δρ_min_ = −0.32 e Å^−^^3^

### Bis(5-amino-1H-tetrazol-4-ium) 5,5'-(3,3'-bi[1,2,4-oxadiazole]-5,5'-diyl)bis(1H-tetrazol-1-olate) (4) Special details

**Table:**

Geometry. All esds (except the esd in the dihedral angle between two l.s. planes) are estimated using the full covariance matrix. The cell esds are taken into account individually in the estimation of esds in distances, angles and torsion angles; correlations between esds in cell parameters are only used when they are defined by crystal symmetry. An approximate (isotropic) treatment of cell esds is used for estimating esds involving l.s. planes.
Refinement. Refinement of F^2^ against ALL reflections. The weighted R-factor wR and goodness of fit S are based on F^2^, conventional R-factors R are based on F, with F set to zero for negative F^2^. The threshold expression of F^2^ > 2sigma(F^2^) is used only for calculating R-factors(gt) etc. and is not relevant to the choice of reflections for refinement. R-factors based on F^2^ are statistically about twice as large as those based on F, and R- factors based on ALL data will be even larger.

### Bis(5-amino-1H-tetrazol-4-ium) 5,5'-(3,3'-bi[1,2,4-oxadiazole]-5,5'-diyl)bis(1H-tetrazol-1-olate) (4) Fractional atomic coordinates and isotropic or equivalent isotropic displacement parameters (Å^2^)

**Table:**

	*x*	*y*	*z*	*U*_iso_*/*U*_eq_	
N1	0.41199 (5)	0.76890 (10)	0.63477 (19)	0.0175 (3)	
N2	0.46216 (6)	0.76711 (11)	0.7314 (2)	0.0222 (3)	
N3	0.47698 (6)	0.66717 (11)	0.7465 (2)	0.0245 (3)	
N4	0.43777 (6)	0.60492 (11)	0.6622 (2)	0.0223 (3)	
C5	0.39732 (6)	0.66827 (12)	0.5933 (2)	0.0170 (3)	
C6	0.34669 (6)	0.63795 (12)	0.4870 (2)	0.0163 (3)	
O7	0.33906 (4)	0.53484 (8)	0.44763 (16)	0.0201 (3)	
N8	0.28771 (6)	0.52728 (10)	0.3395 (2)	0.0207 (3)	
C9	0.27141 (6)	0.62482 (12)	0.3281 (2)	0.0175 (3)	
N10	0.30649 (5)	0.69743 (10)	0.41848 (19)	0.0176 (3)	
C11	0.21877 (6)	0.65335 (12)	0.2242 (2)	0.0169 (3)	
N12	0.20196 (6)	0.75103 (11)	0.2139 (2)	0.0210 (3)	
O13	0.15057 (4)	0.74291 (8)	0.10499 (17)	0.0205 (3)	
C14	0.14345 (6)	0.63995 (12)	0.0628 (2)	0.0170 (3)	
N15	0.18379 (5)	0.58086 (10)	0.1320 (2)	0.0188 (3)	
C16	0.09360 (6)	0.60847 (12)	−0.0484 (2)	0.0176 (3)	
N17	0.07914 (5)	0.50775 (10)	−0.08548 (19)	0.0184 (3)	
N18	0.02984 (6)	0.50893 (11)	−0.1896 (2)	0.0234 (3)	
N19	0.01537 (6)	0.60884 (11)	−0.2111 (2)	0.0233 (3)	
N20	0.05393 (5)	0.67176 (11)	−0.1253 (2)	0.0214 (3)	
O21	0.38511 (5)	0.85499 (9)	0.59296 (17)	0.0235 (3)	
O22	0.10524 (5)	0.42236 (9)	−0.03543 (19)	0.0280 (3)	
C23	−0.12543 (7)	0.63807 (13)	−0.5210 (2)	0.0197 (4)	
N24	−0.12672 (6)	0.53320 (10)	−0.5150 (2)	0.0215 (3)	
H24	−0.100593	0.492838	−0.459091	0.026*	
N25	−0.17470 (6)	0.49815 (12)	−0.6087 (2)	0.0283 (4)	
N26	−0.20249 (6)	0.57784 (11)	−0.6713 (2)	0.0287 (4)	
N27	−0.17293 (6)	0.66536 (11)	−0.6197 (2)	0.0223 (3)	
H27	−0.183456	0.730349	−0.647016	0.027*	
N28	−0.08567 (6)	0.70014 (11)	−0.4466 (2)	0.0270 (4)	
H28A	−0.055908	0.672818	−0.384994	0.032*	
H28B	−0.088925	0.768859	−0.458745	0.032*	
C29	0.62509 (7)	0.63910 (13)	0.9981 (2)	0.0208 (4)	
N30	0.62287 (6)	0.74382 (11)	1.0154 (2)	0.0234 (3)	
H30	0.594969	0.783225	0.971237	0.028*	
N31	0.67036 (6)	0.78048 (12)	1.1116 (2)	0.0269 (4)	
N32	0.70102 (6)	0.70206 (12)	1.1553 (2)	0.0277 (4)	
N33	0.67412 (6)	0.61364 (11)	1.0874 (2)	0.0237 (3)	
H33	0.687071	0.549235	1.100304	0.028*	
N34	0.58793 (6)	0.57604 (11)	0.9108 (2)	0.0272 (4)	
H34A	0.557155	0.602056	0.854686	0.033*	
H34B	0.593824	0.507786	0.908483	0.033*	
O1S	−0.05800 (5)	0.39192 (10)	−0.35612 (19)	0.0270 (3)	
H1SA	−0.0549 (9)	0.328 (2)	−0.374 (3)	0.041*	
H1SB	−0.0263 (9)	0.4142 (18)	−0.305 (3)	0.041*	
O2S	0.71190 (6)	0.42019 (10)	1.1563 (2)	0.0282 (3)	
H2SA	0.7426 (10)	0.4009 (18)	1.192 (3)	0.042*	
H2SB	0.6979 (10)	0.3689 (19)	1.103 (3)	0.042*	
O3S	−0.20626 (6)	0.85908 (10)	−0.6897 (2)	0.0330 (3)	
H3SA	−0.2354 (11)	0.8812 (19)	−0.736 (4)	0.049*	
H3SB	−0.1880 (11)	0.907 (2)	−0.639 (4)	0.049*	
O4S	0.44686 (6)	0.38490 (11)	0.6275 (3)	0.0438 (4)	
H4SA	0.4454 (11)	0.447 (3)	0.636 (4)	0.066*	
H4SB	0.4770 (11)	0.367 (2)	0.674 (4)	0.066*	

### Bis(5-amino-1H-tetrazol-4-ium) 5,5'-(3,3'-bi[1,2,4-oxadiazole]-5,5'-diyl)bis(1H-tetrazol-1-olate) (4) Atomic displacement parameters (Å^2^)

**Table:**

	*U*^11^	*U*^22^	*U*^33^	*U*^12^	*U*^13^	*U*^23^
N1	0.0158 (7)	0.0142 (7)	0.0216 (7)	−0.0010 (5)	−0.0014 (5)	−0.0011 (5)
N2	0.0164 (7)	0.0201 (7)	0.0285 (7)	−0.0001 (6)	−0.0053 (6)	−0.0022 (6)
N3	0.0177 (7)	0.0202 (7)	0.0334 (8)	0.0007 (6)	−0.0069 (6)	−0.0038 (6)
N4	0.0177 (7)	0.0178 (7)	0.0299 (7)	0.0011 (5)	−0.0046 (6)	−0.0017 (6)
C5	0.0150 (8)	0.0144 (8)	0.0210 (8)	−0.0006 (6)	−0.0007 (6)	−0.0003 (6)
C6	0.0153 (8)	0.0122 (7)	0.0212 (8)	−0.0018 (6)	0.0005 (6)	−0.0008 (6)
O7	0.0158 (6)	0.0127 (6)	0.0302 (6)	0.0000 (4)	−0.0054 (5)	−0.0008 (5)
N8	0.0148 (7)	0.0147 (7)	0.0306 (7)	−0.0013 (5)	−0.0072 (6)	−0.0014 (5)
C9	0.0147 (8)	0.0141 (8)	0.0229 (8)	−0.0020 (6)	−0.0017 (6)	−0.0006 (6)
N10	0.0146 (7)	0.0140 (7)	0.0235 (7)	−0.0012 (5)	−0.0019 (5)	−0.0007 (5)
C11	0.0142 (8)	0.0145 (8)	0.0212 (8)	−0.0016 (6)	−0.0012 (6)	−0.0003 (6)
N12	0.0146 (7)	0.0169 (7)	0.0295 (7)	−0.0011 (5)	−0.0077 (6)	−0.0003 (6)
O13	0.0155 (6)	0.0129 (6)	0.0309 (6)	0.0005 (4)	−0.0070 (5)	−0.0029 (5)
C14	0.0160 (8)	0.0123 (7)	0.0222 (8)	−0.0013 (6)	−0.0004 (6)	−0.0006 (6)
N15	0.0159 (7)	0.0130 (7)	0.0264 (7)	−0.0013 (5)	−0.0031 (6)	−0.0008 (5)
C16	0.0155 (8)	0.0121 (7)	0.0245 (8)	−0.0008 (6)	−0.0014 (7)	0.0001 (6)
N17	0.0140 (7)	0.0130 (7)	0.0268 (7)	−0.0002 (5)	−0.0039 (6)	−0.0004 (5)
N18	0.0166 (7)	0.0187 (7)	0.0326 (8)	−0.0010 (6)	−0.0073 (6)	−0.0005 (6)
N19	0.0177 (7)	0.0192 (7)	0.0310 (8)	−0.0008 (6)	−0.0059 (6)	0.0011 (6)
N20	0.0163 (7)	0.0174 (7)	0.0288 (7)	0.0004 (5)	−0.0046 (6)	0.0002 (6)
O21	0.0212 (6)	0.0127 (6)	0.0350 (7)	0.0028 (5)	−0.0046 (5)	0.0003 (5)
O22	0.0229 (7)	0.0109 (6)	0.0471 (8)	0.0019 (5)	−0.0096 (6)	0.0012 (5)
C23	0.0210 (8)	0.0161 (8)	0.0213 (8)	0.0023 (7)	−0.0008 (7)	0.0010 (6)
N24	0.0200 (7)	0.0132 (7)	0.0298 (7)	0.0014 (6)	−0.0043 (6)	0.0018 (6)
N25	0.0233 (8)	0.0194 (8)	0.0402 (8)	−0.0015 (6)	−0.0066 (7)	0.0003 (6)
N26	0.0257 (9)	0.0171 (7)	0.0408 (9)	−0.0009 (6)	−0.0072 (7)	0.0008 (6)
N27	0.0208 (8)	0.0141 (7)	0.0304 (7)	0.0015 (6)	−0.0042 (6)	0.0005 (6)
N28	0.0241 (8)	0.0137 (7)	0.0400 (8)	0.0011 (6)	−0.0104 (7)	0.0025 (6)
C29	0.0214 (9)	0.0175 (8)	0.0231 (8)	0.0032 (7)	0.0008 (7)	0.0005 (6)
N30	0.0214 (8)	0.0164 (7)	0.0311 (8)	0.0027 (6)	−0.0034 (6)	−0.0004 (6)
N31	0.0242 (8)	0.0209 (8)	0.0341 (8)	−0.0001 (6)	−0.0039 (6)	−0.0025 (6)
N32	0.0254 (8)	0.0199 (8)	0.0360 (8)	−0.0010 (6)	−0.0050 (6)	−0.0032 (6)
N33	0.0233 (8)	0.0145 (7)	0.0320 (8)	0.0033 (6)	−0.0033 (6)	−0.0006 (6)
N34	0.0252 (8)	0.0143 (7)	0.0396 (9)	0.0031 (6)	−0.0075 (7)	−0.0013 (6)
O1S	0.0226 (7)	0.0135 (6)	0.0419 (8)	−0.0003 (5)	−0.0104 (6)	−0.0006 (5)
O2S	0.0251 (7)	0.0140 (6)	0.0428 (8)	0.0003 (5)	−0.0089 (6)	−0.0010 (5)
O3S	0.0287 (8)	0.0135 (6)	0.0527 (9)	0.0011 (5)	−0.0136 (6)	−0.0011 (6)
O4S	0.0241 (8)	0.0160 (7)	0.0854 (12)	0.0032 (6)	−0.0204 (8)	−0.0083 (7)

### Bis(5-amino-1H-tetrazol-4-ium) 5,5'-(3,3'-bi[1,2,4-oxadiazole]-5,5'-diyl)bis(1H-tetrazol-1-olate) (4) Geometric parameters (Å, º)

**Table:**

N1—O21	1.2964 (17)	C23—N27	1.337 (2)
N1—N2	1.3429 (19)	N24—N25	1.362 (2)
N1—C5	1.351 (2)	N24—H24	0.8800
N2—N3	1.3230 (19)	N25—N26	1.272 (2)
N3—N4	1.3342 (19)	N26—N27	1.357 (2)
N4—C5	1.331 (2)	N27—H27	0.8800
C5—C6	1.432 (2)	N28—H28A	0.8800
C6—N10	1.297 (2)	N28—H28B	0.8800
C6—O7	1.3468 (18)	C29—N34	1.314 (2)
O7—N8	1.4033 (16)	C29—N30	1.338 (2)
N8—C9	1.303 (2)	C29—N33	1.338 (2)
C9—N10	1.368 (2)	N30—N31	1.366 (2)
C9—C11	1.461 (2)	N30—H30	0.8800
C11—N12	1.309 (2)	N31—N32	1.269 (2)
C11—N15	1.3704 (19)	N32—N33	1.362 (2)
N12—O13	1.4071 (17)	N33—H33	0.8800
O13—C14	1.3473 (18)	N34—H34A	0.8800
C14—N15	1.298 (2)	N34—H34B	0.8800
C14—C16	1.435 (2)	O1S—H1SA	0.83 (3)
C16—N20	1.333 (2)	O1S—H1SB	0.87 (2)
C16—N17	1.346 (2)	O2S—H2SA	0.81 (2)
N17—O22	1.2902 (17)	O2S—H2SB	0.81 (3)
N17—N18	1.3450 (19)	O3S—H3SA	0.81 (2)
N18—N19	1.3232 (19)	O3S—H3SB	0.82 (3)
N19—N20	1.3322 (19)	O4S—H4SA	0.79 (3)
C23—N28	1.320 (2)	O4S—H4SB	0.81 (3)
C23—N24	1.334 (2)		
			
O21—N1—N2	123.26 (12)	N18—N19—N20	110.74 (13)
O21—N1—C5	129.24 (13)	N19—N20—C16	105.92 (13)
N2—N1—C5	107.49 (12)	N28—C23—N24	127.21 (15)
N3—N2—N1	106.79 (12)	N28—C23—N27	128.20 (15)
N2—N3—N4	110.65 (13)	N24—C23—N27	104.59 (14)
C5—N4—N3	106.20 (13)	C23—N24—N25	109.56 (13)
N4—C5—N1	108.87 (14)	C23—N24—H24	125.2
N4—C5—C6	126.93 (14)	N25—N24—H24	125.2
N1—C5—C6	124.17 (14)	N26—N25—N24	108.08 (14)
N10—C6—O7	114.16 (13)	N25—N26—N27	107.94 (14)
N10—C6—C5	128.49 (14)	C23—N27—N26	109.82 (14)
O7—C6—C5	117.34 (13)	C23—N27—H27	125.1
C6—O7—N8	105.75 (11)	N26—N27—H27	125.1
C9—N8—O7	102.93 (12)	C23—N28—H28A	120.0
N8—C9—N10	115.91 (14)	C23—N28—H28B	120.0
N8—C9—C11	121.30 (14)	H28A—N28—H28B	120.0
N10—C9—C11	122.79 (14)	N34—C29—N30	127.83 (16)
C6—N10—C9	101.26 (13)	N34—C29—N33	128.02 (15)
N12—C11—N15	115.54 (14)	N30—C29—N33	104.15 (14)
N12—C11—C9	121.58 (14)	C29—N30—N31	109.89 (14)
N15—C11—C9	122.88 (14)	C29—N30—H30	125.1
C11—N12—O13	102.91 (12)	N31—N30—H30	125.1
C14—O13—N12	105.92 (11)	N32—N31—N30	107.93 (14)
N15—C14—O13	113.96 (13)	N31—N32—N33	108.03 (14)
N15—C14—C16	128.05 (14)	C29—N33—N32	110.00 (14)
O13—C14—C16	117.99 (13)	C29—N33—H33	125.0
C14—N15—C11	101.67 (13)	N32—N33—H33	125.0
N20—C16—N17	109.25 (13)	C29—N34—H34A	120.0
N20—C16—C14	126.58 (14)	C29—N34—H34B	120.0
N17—C16—C14	124.14 (14)	H34A—N34—H34B	120.0
O22—N17—N18	123.35 (13)	H1SA—O1S—H1SB	106 (2)
O22—N17—C16	129.37 (13)	H2SA—O2S—H2SB	104 (2)
N18—N17—C16	107.28 (12)	H3SA—O3S—H3SB	110 (2)
N19—N18—N17	106.80 (12)	H4SA—O4S—H4SB	107 (3)
			
O21—N1—N2—N3	−179.48 (14)	O13—C14—N15—C11	−0.16 (18)
C5—N1—N2—N3	0.14 (17)	C16—C14—N15—C11	−179.50 (16)
N1—N2—N3—N4	0.05 (19)	N12—C11—N15—C14	−0.14 (19)
N2—N3—N4—C5	−0.22 (19)	C9—C11—N15—C14	179.76 (15)
N3—N4—C5—N1	0.30 (18)	N15—C14—C16—N20	−176.68 (16)
N3—N4—C5—C6	178.57 (15)	O13—C14—C16—N20	4.0 (2)
O21—N1—C5—N4	179.30 (15)	N15—C14—C16—N17	5.5 (3)
N2—N1—C5—N4	−0.28 (18)	O13—C14—C16—N17	−173.77 (15)
O21—N1—C5—C6	1.0 (3)	N20—C16—N17—O22	−179.06 (15)
N2—N1—C5—C6	−178.60 (14)	C14—C16—N17—O22	−0.9 (3)
N4—C5—C6—N10	179.82 (16)	N20—C16—N17—N18	0.78 (18)
N1—C5—C6—N10	−2.2 (3)	C14—C16—N17—N18	178.89 (15)
N4—C5—C6—O7	−1.6 (2)	O22—N17—N18—N19	179.15 (14)
N1—C5—C6—O7	176.45 (14)	C16—N17—N18—N19	−0.70 (18)
N10—C6—O7—N8	0.35 (18)	N17—N18—N19—N20	0.38 (18)
C5—C6—O7—N8	−178.46 (13)	N18—N19—N20—C16	0.10 (18)
C6—O7—N8—C9	−0.01 (16)	N17—C16—N20—N19	−0.54 (18)
O7—N8—C9—N10	−0.31 (18)	C14—C16—N20—N19	−178.59 (15)
O7—N8—C9—C11	179.62 (14)	N28—C23—N24—N25	−179.78 (17)
O7—C6—N10—C9	−0.50 (18)	N27—C23—N24—N25	−0.22 (18)
C5—C6—N10—C9	178.15 (16)	C23—N24—N25—N26	0.0 (2)
N8—C9—N10—C6	0.51 (19)	N24—N25—N26—N27	0.2 (2)
C11—C9—N10—C6	−179.43 (15)	N28—C23—N27—N26	179.88 (17)
N8—C9—C11—N12	179.34 (16)	N24—C23—N27—N26	0.33 (18)
N10—C9—C11—N12	−0.7 (2)	N25—N26—N27—C23	−0.3 (2)
N8—C9—C11—N15	−0.6 (2)	N34—C29—N30—N31	−178.26 (17)
N10—C9—C11—N15	179.38 (15)	N33—C29—N30—N31	0.79 (18)
N15—C11—N12—O13	0.36 (18)	C29—N30—N31—N32	−0.74 (19)
C9—C11—N12—O13	−179.54 (14)	N30—N31—N32—N33	0.35 (19)
C11—N12—O13—C14	−0.42 (16)	N34—C29—N33—N32	178.47 (17)
N12—O13—C14—N15	0.38 (18)	N30—C29—N33—N32	−0.58 (19)
N12—O13—C14—C16	179.79 (13)	N31—N32—N33—C29	0.1 (2)

### Bis(5-amino-1H-tetrazol-4-ium) 5,5'-(3,3'-bi[1,2,4-oxadiazole]-5,5'-diyl)bis(1H-tetrazol-1-olate) (4) Hydrogen-bond geometry (Å, º)

**Table:**

*D*—H···*A*	*D*—H	H···*A*	*D*···*A*	*D*—H···*A*
N24—H24···O1*S*	0.88	1.76	2.6267 (18)	169
N27—H27···O3*S*	0.88	1.74	2.6241 (19)	177
N28—H28*A*···N19	0.88	2.18	3.054 (2)	176
N28—H28*B*···O22^i^	0.88	1.99	2.8656 (19)	172
N30—H30···O4*S*^ii^	0.88	1.75	2.605 (2)	165
N33—H33···O2*S*	0.88	1.78	2.6544 (19)	173
N34—H34*A*···N3	0.88	2.20	3.080 (2)	174
N34—H34*B*···O21^iii^	0.88	2.01	2.8882 (19)	175
O1*S*—H1*SA*···N20^iv^	0.83 (3)	1.99 (3)	2.8030 (19)	170 (2)
O1*S*—H1*SB*···N18	0.87 (2)	1.94 (2)	2.7758 (19)	160 (2)
O2*S*—H2*SA*···N12^iii^	0.81 (2)	2.39 (2)	3.0906 (18)	144 (2)
O2*S*—H2*SB*···N10^iii^	0.81 (3)	2.19 (2)	2.9038 (18)	148 (2)
O3*S*—H3*SA*···N8^i^	0.81 (2)	2.33 (2)	3.0397 (19)	147 (2)
O3*S*—H3*SB*···N15^i^	0.82 (3)	2.21 (3)	2.8915 (19)	141 (2)
O4*S*—H4*SA*···N4	0.79 (3)	2.03 (3)	2.817 (2)	177 (3)
O4*S*—H4*SB*···N2^iii^	0.81 (3)	2.02 (3)	2.789 (2)	157 (3)

Symmetry codes: (i) −*x*, *y*+1/2, −*z*−1/2; (ii) −*x*+1, *y*+1/2, −*z*+3/2; (iii) −*x*+1, *y*−1/2, −*z*+3/2; (iv) −*x*, *y*−1/2, −*z*−1/2.

### Bis(aminoguanidinium) 5,5'-(3,3'-bi[1,2,4-oxadiazole]-5,5'-diyl)bis(1H-tetrazol-1-olate) (5) Crystal data

**Table:**

2CH_7_N_4_^+^·C_6_N_12_O_4_^2^^−^	*F*(000) = 468
*M**_r_* = 454.39	*D*_x_ = 1.673 Mg m^−^^3^
Monoclinic, *P*2_1_/*n*	Mo *K*α radiation, λ = 0.71073 Å
*a* = 7.9458 (4) Å	Cell parameters from 4111 reflections
*b* = 5.5586 (2) Å	θ = 2.9–26.4°
*c* = 20.6066 (9) Å	µ = 0.14 mm^−^^1^
β = 97.647 (2)°	*T* = 150 K
*V* = 902.05 (7) Å^3^	Rod, yellow
*Z* = 2	0.42 × 0.11 × 0.08 mm

### Bis(aminoguanidinium) 5,5'-(3,3'-bi[1,2,4-oxadiazole]-5,5'-diyl)bis(1H-tetrazol-1-olate) (5) Data collection

**Table:**

Bruker SMART APEXII CCD diffractometer	1633 reflections with *I* > 2σ(*I*)
Radiation source: fine focus sealed tube	*R*_int_ = 0.020
ω scans	θ_max_ = 26.4°, θ_min_ = 2.7°
Absorption correction: multi-scan (SADABS; Bruker, 2014)	*h* = −9→9
*T*_min_ = 0.944, *T*_max_ = 0.989	*k* = −6→6
7786 measured reflections	*l* = −25→25
1844 independent reflections	

### Bis(aminoguanidinium) 5,5'-(3,3'-bi[1,2,4-oxadiazole]-5,5'-diyl)bis(1H-tetrazol-1-olate) (5) Refinement

**Table:**

Refinement on *F*^2^	63 restraints
Least-squares matrix: full	Hydrogen site location: mixed
*R*[*F*^2^ > 2σ(*F*^2^)] = 0.045	H atoms treated by a mixture of independent and constrained refinement
*wR*(*F*^2^) = 0.134	*w* = 1/[σ^2^(*F*_o_^2^) + (0.0711*P*)^2^ + 0.8763*P*] where *P* = (*F*_o_^2^ + 2*F*_c_^2^)/3
*S* = 1.06	(Δ/σ)_max_ < 0.001
1844 reflections	Δρ_max_ = 0.74 e Å^−^^3^
206 parameters	Δρ_min_ = −0.24 e Å^−^^3^

### Bis(aminoguanidinium) 5,5'-(3,3'-bi[1,2,4-oxadiazole]-5,5'-diyl)bis(1H-tetrazol-1-olate) (5) Special details

**Table:**

Geometry. All esds (except the esd in the dihedral angle between two l.s. planes) are estimated using the full covariance matrix. The cell esds are taken into account individually in the estimation of esds in distances, angles and torsion angles; correlations between esds in cell parameters are only used when they are defined by crystal symmetry. An approximate (isotropic) treatment of cell esds is used for estimating esds involving l.s. planes.
Refinement. Refinement of F^2^ against ALL reflections. The weighted R-factor wR and goodness of fit S are based on F^2^, conventional R-factors R are based on F, with F set to zero for negative F^2^. The threshold expression of F^2^ > 2sigma(F^2^) is used only for calculating R-factors(gt) etc. and is not relevant to the choice of reflections for refinement. R-factors based on F^2^ are statistically about twice as large as those based on F, and R- factors based on ALL data will be even larger.

### Bis(aminoguanidinium) 5,5'-(3,3'-bi[1,2,4-oxadiazole]-5,5'-diyl)bis(1H-tetrazol-1-olate) (5) Fractional atomic coordinates and isotropic or equivalent isotropic displacement parameters (Å^2^)

**Table:**

	*x*	*y*	*z*	*U*_iso_*/*U*_eq_	Occ. (<1)
N1A	0.7242 (6)	0.6920 (8)	0.89476 (16)	0.0200 (8)	0.907 (5)
N2A	0.7542 (4)	0.8420 (6)	0.84668 (15)	0.0249 (7)	0.907 (5)
N3A	0.6683 (3)	0.7570 (6)	0.79207 (13)	0.0273 (7)	0.907 (5)
N4A	0.5827 (5)	0.5580 (6)	0.80370 (19)	0.0256 (8)	0.907 (5)
C5A	0.6185 (8)	0.5198 (9)	0.8676 (2)	0.0197 (10)	0.907 (5)
O11A	0.7863 (2)	0.7262 (3)	0.95566 (8)	0.0277 (5)	0.907 (5)
N1B	0.599 (6)	0.619 (7)	0.809 (2)	0.025 (7)	0.093 (5)
N2B	0.685 (4)	0.813 (5)	0.8096 (18)	0.019 (5)	0.093 (5)
N3B	0.774 (5)	0.878 (6)	0.8619 (17)	0.021 (5)	0.093 (5)
N4B	0.744 (6)	0.696 (10)	0.909 (2)	0.020 (6)	0.093 (5)
C5B	0.635 (10)	0.536 (10)	0.878 (3)	0.023 (8)	0.093 (5)
O11B	0.483 (2)	0.528 (3)	0.7672 (8)	0.030 (5)	0.093 (5)
C6	0.5571 (2)	0.3235 (3)	0.90328 (9)	0.0191 (4)	
O7	0.44405 (17)	0.1736 (2)	0.86989 (6)	0.0226 (3)	
N8	0.4063 (2)	−0.0012 (3)	0.91550 (8)	0.0222 (4)	
C9	0.5000 (2)	0.0668 (3)	0.96935 (8)	0.0192 (4)	
N10	0.5969 (2)	0.2681 (3)	0.96468 (7)	0.0203 (4)	
N12	0.1089 (3)	0.6386 (5)	0.79393 (10)	0.0481 (6)	
H12A	0.167 (3)	0.578 (5)	0.7645 (10)	0.058*	
H12B	0.029 (3)	0.723 (5)	0.7706 (13)	0.058*	
N13	0.0329 (3)	0.4421 (4)	0.82311 (9)	0.0403 (5)	
H13	−0.0403	0.3478	0.7995	0.048*	
C14	0.0749 (2)	0.4033 (4)	0.88724 (10)	0.0276 (5)	
N15	0.0053 (3)	0.2218 (4)	0.91527 (10)	0.0387 (5)	
H15A	0.0311	0.1965	0.9576	0.046*	
H15B	−0.0670	0.1262	0.8917	0.046*	
N16	0.1827 (2)	0.5488 (3)	0.92093 (8)	0.0296 (4)	
H16A	0.2103	0.5268	0.9633	0.036*	
H16B	0.2276	0.6687	0.9012	0.036*	

### Bis(aminoguanidinium) 5,5'-(3,3'-bi[1,2,4-oxadiazole]-5,5'-diyl)bis(1H-tetrazol-1-olate) (5) Atomic displacement parameters (Å^2^)

**Table:**

	*U*^11^	*U*^22^	*U*^33^	*U*^12^	*U*^13^	*U*^23^
N1A	0.0220 (15)	0.0203 (11)	0.0168 (18)	0.0009 (10)	−0.0010 (12)	−0.0005 (13)
N2A	0.0294 (15)	0.0221 (14)	0.0221 (17)	−0.0029 (10)	−0.0001 (14)	0.0003 (11)
N3A	0.0349 (14)	0.0276 (14)	0.0182 (13)	−0.0039 (10)	−0.0012 (10)	0.0013 (10)
N4A	0.0295 (18)	0.0255 (17)	0.0210 (13)	−0.0052 (12)	0.0004 (11)	0.0027 (12)
C5A	0.019 (2)	0.0215 (13)	0.018 (2)	−0.0001 (11)	0.0004 (15)	−0.0022 (12)
O11A	0.0332 (9)	0.0290 (9)	0.0185 (10)	−0.0056 (7)	−0.0054 (7)	−0.0019 (6)
N1B	0.030 (11)	0.023 (9)	0.019 (7)	−0.006 (8)	−0.006 (6)	0.008 (5)
N2B	0.021 (6)	0.019 (6)	0.017 (6)	−0.002 (3)	0.004 (3)	0.003 (3)
N3B	0.022 (6)	0.021 (6)	0.021 (6)	0.001 (3)	0.001 (3)	0.001 (3)
N4B	0.020 (11)	0.022 (8)	0.017 (8)	−0.006 (7)	−0.002 (6)	0.005 (6)
C5B	0.026 (14)	0.027 (9)	0.013 (8)	−0.005 (10)	−0.004 (7)	0.007 (6)
O11B	0.032 (7)	0.036 (8)	0.020 (7)	−0.008 (6)	−0.001 (6)	0.002 (5)
C6	0.0170 (8)	0.0210 (9)	0.0186 (8)	0.0006 (7)	0.0000 (6)	−0.0025 (7)
O7	0.0251 (7)	0.0244 (7)	0.0172 (6)	−0.0043 (6)	−0.0015 (5)	−0.0004 (5)
N8	0.0256 (8)	0.0233 (8)	0.0173 (7)	−0.0040 (6)	0.0010 (6)	0.0001 (6)
C9	0.0196 (8)	0.0203 (9)	0.0174 (9)	−0.0008 (7)	0.0015 (6)	−0.0019 (7)
N10	0.0217 (8)	0.0215 (8)	0.0174 (8)	−0.0020 (6)	0.0008 (6)	−0.0009 (6)
N12	0.0518 (13)	0.0630 (16)	0.0285 (10)	0.0028 (12)	0.0017 (9)	0.0059 (10)
N13	0.0441 (11)	0.0451 (12)	0.0282 (10)	−0.0039 (9)	−0.0081 (8)	−0.0064 (9)
C14	0.0225 (9)	0.0272 (11)	0.0319 (11)	0.0016 (8)	−0.0007 (8)	−0.0078 (9)
N15	0.0361 (10)	0.0349 (11)	0.0430 (11)	−0.0086 (8)	−0.0031 (9)	−0.0020 (9)
N16	0.0304 (9)	0.0324 (10)	0.0241 (8)	−0.0078 (8)	−0.0037 (7)	−0.0027 (7)

### Bis(aminoguanidinium) 5,5'-(3,3'-bi[1,2,4-oxadiazole]-5,5'-diyl)bis(1H-tetrazol-1-olate) (5) Geometric parameters (Å, º)

**Table:**

N1A—O11A	1.300 (3)	O7—N8	1.411 (2)
N1A—N2A	1.341 (5)	N8—C9	1.307 (2)
N1A—C5A	1.345 (5)	C9—N10	1.369 (2)
N2A—N3A	1.323 (3)	C9—C9^i^	1.465 (4)
N3A—N4A	1.337 (4)	N12—N13	1.420 (3)
N4A—C5A	1.328 (5)	N12—H12A	0.8800 (11)
C5A—C6	1.436 (4)	N12—H12B	0.8801 (11)
N1B—N2B	1.28 (5)	N13—C14	1.337 (3)
N1B—O11B	1.28 (4)	N13—H13	0.8800
N1B—C5B	1.48 (7)	C14—N16	1.309 (3)
N2B—N3B	1.26 (4)	C14—N15	1.320 (3)
N3B—N4B	1.45 (6)	N15—H15A	0.8800
N4B—C5B	1.34 (7)	N15—H15B	0.8800
C5B—C6	1.46 (2)	N16—H16A	0.8800
C6—N10	1.300 (2)	N16—H16B	0.8800
C6—O7	1.345 (2)		
			
O11A—N1A—N2A	122.7 (4)	O7—C6—C5B	127 (2)
O11A—N1A—C5A	129.9 (4)	C6—O7—N8	105.81 (13)
N2A—N1A—C5A	107.4 (3)	C9—N8—O7	102.65 (14)
N3A—N2A—N1A	106.5 (3)	N8—C9—N10	116.01 (16)
N2A—N3A—N4A	111.1 (3)	N8—C9—C9^i^	121.4 (2)
C5A—N4A—N3A	105.3 (2)	N10—C9—C9^i^	122.6 (2)
N4A—C5A—N1A	109.8 (3)	C6—N10—C9	101.30 (15)
N4A—C5A—C6	125.9 (3)	N13—N12—H12A	107 (2)
N1A—C5A—C6	124.3 (4)	N13—N12—H12B	109 (2)
N2B—N1B—O11B	132 (4)	H12A—N12—H12B	104 (3)
N2B—N1B—C5B	103 (3)	C14—N13—N12	118.59 (19)
O11B—N1B—C5B	124 (4)	C14—N13—H13	120.7
N3B—N2B—N1B	119 (3)	N12—N13—H13	120.7
N2B—N3B—N4B	105 (3)	N16—C14—N15	121.5 (2)
C5B—N4B—N3B	107 (4)	N16—C14—N13	119.0 (2)
N4B—C5B—C6	129 (5)	N15—C14—N13	119.5 (2)
N4B—C5B—N1B	107 (3)	C14—N15—H15A	120.0
C6—C5B—N1B	124 (4)	C14—N15—H15B	120.0
N10—C6—O7	114.22 (17)	H15A—N15—H15B	120.0
N10—C6—C5A	128.5 (2)	C14—N16—H16A	120.0
O7—C6—C5A	117.3 (2)	C14—N16—H16B	120.0
N10—C6—C5B	119 (2)	H16A—N16—H16B	120.0
			
O11A—N1A—N2A—N3A	−178.2 (4)	N1A—C5A—C6—N10	−4.2 (8)
C5A—N1A—N2A—N3A	−0.6 (5)	N4A—C5A—C6—O7	−4.8 (8)
N1A—N2A—N3A—N4A	0.6 (4)	N1A—C5A—C6—O7	176.2 (5)
N2A—N3A—N4A—C5A	−0.3 (5)	N4B—C5B—C6—N10	−2 (10)
N3A—N4A—C5A—N1A	0.0 (6)	N1B—C5B—C6—N10	−179 (5)
N3A—N4A—C5A—C6	−179.2 (5)	N4B—C5B—C6—O7	177 (6)
O11A—N1A—C5A—N4A	177.8 (5)	N1B—C5B—C6—O7	0 (10)
N2A—N1A—C5A—N4A	0.4 (6)	N10—C6—O7—N8	−0.1 (2)
O11A—N1A—C5A—C6	−3.0 (9)	C5A—C6—O7—N8	179.5 (4)
N2A—N1A—C5A—C6	179.6 (5)	C5B—C6—O7—N8	−180 (5)
O11B—N1B—N2B—N3B	170 (5)	C6—O7—N8—C9	0.14 (18)
C5B—N1B—N2B—N3B	2 (6)	O7—N8—C9—N10	−0.1 (2)
N1B—N2B—N3B—N4B	−2 (5)	O7—N8—C9—C9^i^	179.9 (2)
N2B—N3B—N4B—C5B	1 (6)	O7—C6—N10—C9	0.0 (2)
N3B—N4B—C5B—C6	−177 (7)	C5A—C6—N10—C9	−179.6 (4)
N3B—N4B—C5B—N1B	0 (7)	C5B—C6—N10—C9	180 (4)
N2B—N1B—C5B—N4B	−1 (7)	N8—C9—N10—C6	0.1 (2)
O11B—N1B—C5B—N4B	−170 (5)	C9^i^—C9—N10—C6	−180.0 (2)
N2B—N1B—C5B—C6	176 (6)	N12—N13—C14—N16	0.8 (3)
O11B—N1B—C5B—C6	7 (10)	N12—N13—C14—N15	179.8 (2)
N4A—C5A—C6—N10	174.8 (5)		

Symmetry code: (i) −*x*+1, −*y*, −*z*+2.

### Bis(aminoguanidinium) 5,5'-(3,3'-bi[1,2,4-oxadiazole]-5,5'-diyl)bis(1H-tetrazol-1-olate) (5) Hydrogen-bond geometry (Å, º)

**Table:**

*D*—H···*A*	*D*—H	H···*A*	*D*···*A*	*D*—H···*A*
N12—H12*B*···N4*A*^ii^	0.88 (1)	2.50 (1)	3.314 (4)	155 (3)
N13—H13···N3*A*^iii^	0.88	2.08	2.870 (3)	149
N13—H13···N4*A*^iii^	0.88	2.65	3.405 (4)	144
N15—H15*A*···O11*A*^iv^	0.88	2.19	2.954 (3)	145
N15—H15*B*···N2*A*^v^	0.88	2.24	3.112 (4)	170
N16—H16*A*···O11*A*^iv^	0.88	2.18	2.949 (2)	146
N16—H16*A*···N10^iv^	0.88	2.29	2.926 (2)	129
N16—H16*B*···N8^vi^	0.88	2.32	3.079 (2)	145

Symmetry codes: (ii) −*x*+1/2, *y*+1/2, −*z*+3/2; (iii) −*x*+1/2, *y*−1/2, −*z*+3/2; (iv) −*x*+1, −*y*+1, −*z*+2; (v) *x*−1, *y*−1, *z*; (vi) *x*, *y*+1, *z*.

## References

[bb1] Akella, A. & Keszler, D. A. (1994). *Acta Cryst.* C**50**, 1974–1976.

[bb14] Akhavan, J. (2011). *The Chemistry of Explosives*, pp. 68–69. Cambridge: RSC Publishing.

[bb2] Allen, F. H., Kennard, O., Watson, D. G., Brammer, L., Orpen, A. G. & Taylor, R. (1987). *J. Chem. Soc. Perkin Trans. 2*, pp. S1–S19.

[bb3] Bruker (2010). *APEX2*. Bruker AXS Inc., Madison, Wisconsin, USA.

[bb4] Bruker (2014). *SAINT*, *XPREP* and *SADABS*. Bruker AXS Inc., Madison, Wisconsin, USA.

[bb5] Denffer, M. von, Klapötke, T. M., Kramer, G., Spiess, G., Welch, J. M. & Heeb, G. (2005). *Propellants, Explosives, Pyrotech.* **30**, 191–195.

[bb6] Dickens, B. (1969). *Acta Cryst.* B**25**, 1875–1882.

[bb7] Groom, C. R., Bruno, I. J., Lightfoot, M. P. & Ward, S. C. (2016). *Acta Cryst.* B**72**, 171–179. 10.1107/S2052520616003954 PMC4822653 27048719

[bb8] Ma, Y., Zhang, A., Zhang, C., Jiang, D., Zhu, Y. & Zhang, C. (2014). *Cryst. Growth Des.* **14**, 4703–4713.

[bb9] Pagoria, P. F., Zhang, M. X., Zuckerman, N. B., DeHope, A. J. & Parrish, D. A. (2017). *Chem. Heterocycl. C.* **53**, 760–778.

[bb10] Sakurai, K. & Tomiie, Y. (1952). *Acta Cryst.* **5**, 293–294.

[bb11] Schottel, B. L., Chifotides, H. T. & Dunbar, K. R. (2008). *Chem. Soc. Rev.* **37**, 68–83. 10.1039/b614208g 18197334

[bb12] Sheldrick, G. M. (2008). *Acta Cryst.* A**64**, 112–122. 10.1107/S0108767307043930 18156677

[bb13] Sheldrick, G. M. (2015). *Acta Cryst.* C**71**, 3–8.

